# Proteomic analysis of cortical neuronal cultures treated with poly-arginine peptide-18 (R18) and exposed to glutamic acid excitotoxicity

**DOI:** 10.1186/s13041-019-0486-8

**Published:** 2019-07-17

**Authors:** Gabriella MacDougall, Ryan S. Anderton, Frank L. Mastaglia, Neville W. Knuckey, Bruno P. Meloni

**Affiliations:** 1 Centre for Neuromuscular and Neurological Disorders, The University of Western Australia, Nedlands, Australia; 2 Department of Neurosurgery, Sir Charles Gairdner Hospital, QEII Medical Centre, Nedlands, Western Australia Australia; 3 Perron Institute for Neurological and Translational Sciences, QEII Medical Centre, Ground Floor, RR Block, 8 Verdun St, Nedlands, Western Australia 6009 Australia; 4 School of Heath Sciences and Institute for Health Research, The University Notre Dame, Fremantle, Western Australia Australia

**Keywords:** Poly-arginine-18 (R18), iTRAQ proteomics, Neuroprotection, Mito-protection, Excitotoxicity, Stroke

## Abstract

**Abstract:**

Poly-arginine peptide-18 (R18) has recently emerged as a highly effective neuroprotective agent in experimental stroke models, and is particularly efficacious in protecting cortical neurons against glutamic acid excitotoxicity. While we have previously demonstrated that R18 can reduce excitotoxicity-induced neuronal calcium influx, other molecular events associated with R18 neuroprotection are yet to investigated. Therefore, in this study we were particularly interested in protein expression changes in R18 treated neurons subjected to excitotoxicity.

Proteomic analysis was used to compare protein expression patterns in primary cortical neuronal cultures subjected to: (i) R18-treatment alone (R18); (ii) glutamic acid excitotoxic injury (Glut); (iii) R18-treatment and glutamic acid injury (R18 + Glut); (iv) no treatment (Cont). Whole cell lysates were harvested 24 h post-injury and subjected to quantitative proteomic analysis (iTRAQ), coupled with liquid chromatography-tandem mass spectrometry (LC-MS/MS) and subsequent bioinformatic analysis of differentially expressed proteins (DEPs).

Relative to control cultures, R18, Glut, and R18 + Glut treatment resulted in the detection of 5, 95 and 14 DEPs respectively. Compared to Glut alone, R18 + Glut revealed 98 DEPs, including 73 proteins whose expression was also altered by treatment with Glut and/or R18 alone, as well as 25 other uniquely regulated proteins. R18 treatment reversed the up- or down-regulation of all 73 Glut-associated DEPs, which included proteins involved in mitochondrial integrity, ATP generation, mRNA processing and protein translation. Analysis of protein-protein interactions of the 73 DEPs showed they were primarily associated with mitochondrial respiration, proteasome activity and protein synthesis, transmembrane trafficking, axonal growth and neuronal differentiation, and carbohydrate metabolism. Identified protein pathways associated with proteostasis and energy metabolism, and with pathways involved in neurodegeneration.

Collectively, the findings indicate that R18 neuroprotection following excitotoxicity is associated with preservation of neuronal protein profiles, and differential protein expression that assists in maintaining mitochondrial function and energy production, protein homeostasis, and membrane trafficking.

**Graphical abstract:**

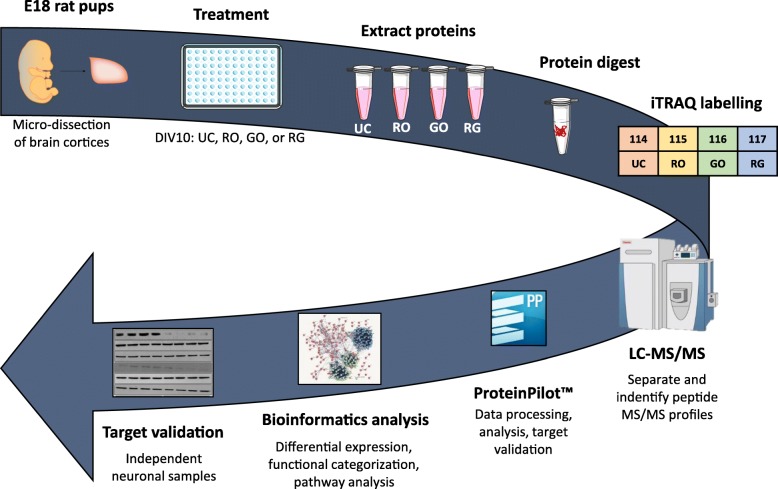

**Electronic supplementary material:**

The online version of this article (10.1186/s13041-019-0486-8) contains supplementary material, which is available to authorized users.

## Introduction

A major pathophysiological mechanism responsible for ischaemic stroke injury is excitotoxicity, which is trigged by the excessive release of the excitatory neurotransmitter glutamic acid in response to reduced cerebral blood flow and compromised ATP synthesis. Excitotoxicity initiates a range of forward-feeding biochemical events known as the ‘ischaemic cascade’, which if not inhibited eventually lead to neuronal death and cerebral infarction [1]. Furthermore, as glutamic acid is the most prominent excitatory neurotransmitter in the CNS [2], the detrimental effects of glutamic acid excitotoxicity also play a role in other acute brain disorders such as traumatic brain injury and epilepsy, as well as chronic neurodegenerative disorders, such as Alzheimer’s disease (AD) [3, 4], Huntington’s disease (HD) [5, 6], Parkinson’s disease (PD) [7, 8], and amyotrophic lateral sclerosis (ALS) [9, 10].

Despite ongoing research, neuroprotective therapies for acute brain injuries and other neurodegenerative disorders are either not available or are extremely limited with modest efficacy. Recent studies in our laboratory have identified cationic arginine-rich peptides (CARPs), which include poly-arginine peptides, as a novel class of neuroprotective agents. In particular, we have demonstrated that poly-arginine-18 (R18, 18-mer of arginine) is neuroprotective in in vitro neuronal excitotoxicity models and in vivo in rodent models of stroke [11–18], hypoxic-ischaemic encephalopathy (HIE) [19], and traumatic brain injury (TBI) [20, 21].

**Fig. 1:**
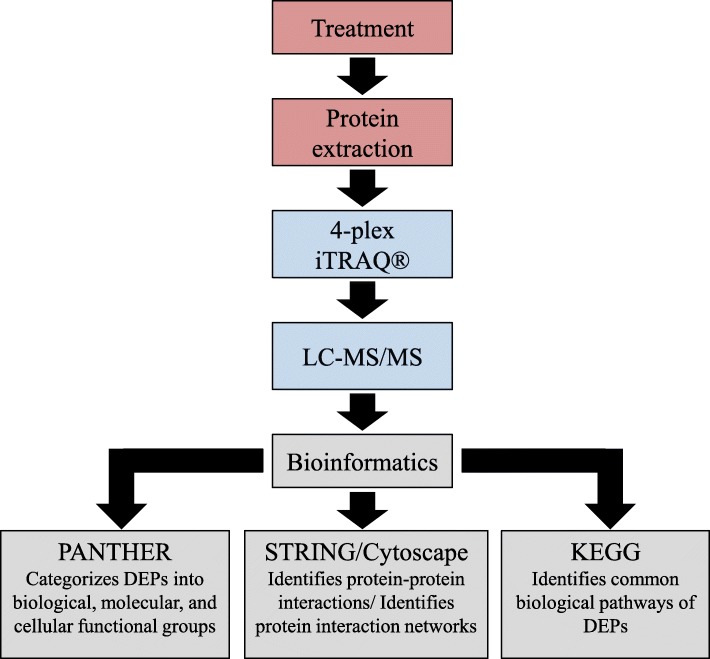
Summary diagram of experimental flow, with the three key processes highlighted by different colours. These broadly include the initial cell treatment and protein collection (red); protein processing, purification, iTRAQ labelling, and quantification for each sample (blue); and analysis of bioinformatics data (grey). DEPs = differentially expressed proteins; LC-MS/MS = liquid chromatography-tandem mass spectrometry

Given the neuroprotective properties of R18, it is imperative that the molecular pathways that underlie its neuroprotective action are fully elucidated in order to gauge its therapeutic potential. While we have previously demonstrated that R18 has the capacity to reduce glutamic acid-induced excitotoxic neuronal death and intracellular calcium influx, and reduce neuronal NMDA receptor levels [22], CARPs also have cell-penetrating properties and can target mitochondria [23]. Therefore, it is likely that R18 and other CARPs have additional intracellular neuroprotective mechanisms of action. In addition, it is also important to examine the ability of R18 to preserve intracellular protein expression and biochemical pathways following a neurodamaging insult. As such, in this study we performed iTRAQ proteomics and bioinformatic analysis (Fig. 1) of protein cell lysates collected from primary cortical neuronal cultures subjected to glutamic acid excitotoxicity with and without treatment with R18.

## Methods

### Peptides

Poly-arginine-18 (R18; H-RRRRRRRRRRRRRRRRRR-OH) was synthesized by Mimotopes (Australia) and purified to 98% by HPLC. Peptides were prepared as 500 μM stocks in Baxter water (Australia) and stored at − 20 °C prior to use.

### Primary cortical neuronal cultures

Cortical neuronal tissue was extracted from E18 Sprague-Dawley rat embryos, dissociated, resuspended in Neurobasal/2% B27 supplement (B27) and seeded at approximately 55,000 cells/well into 96-well plates (Nunc, Australia), pre-coated with poly-lysine (Sigma-Aldrich Australia) as previously described [24]. Plates were maintained at 37 °C in a CO_2_ incubator (95% air balance, 98% humidity, 5% CO_2_) until use on day in vitro 10, when cultures routinely comprise > 97% neurons and 1–3% astrocytes. Approval for the use of E18 Sprague-Dawley rat embryos for isolation of cortical tissue was obtained by the University of Western Australia Animal Ethics Committee (RA/3/100/1432).

### Glutamic acid excitotoxicity model and assessment of cell viability

Cortical neuronal cultures were subjected to glutamic acid excitotoxicity and R18 treatment as previously described [22]. R18 treatment consisted of adding the peptide to culture wells 10 min prior to glutamic acid (L-glutamic acid; Sigma-Aldrich) exposure by removing media and adding 50 μL of Minimal Essential Media (MEM)/2% B27 containing peptide (2 μM). To induce excitotoxicity, 50 μL of MEM/2% B27 containing glutamic acid (200 μM; final concentration 100 μM) was added to the culture wells and incubated at 37 °C in the CO_2_ incubator for 5 min (note: peptide concentration reduced to 1 μM during this step). Following the 5-min exposure, media was replaced with 100 μL of MEM/2% B27 and cultures incubated for a further 24 h at 37 °C in the CO_2_ incubator. Untreated controls with or without glutamic acid treatment underwent the same incubation steps and media additions.

At 24 h post-injury, cell viability was assessed qualitatively by light microscopy, and quantitatively using the CellTiter 96 Aqueous Cell Proliferation MTS assay (Promega, Australia), which determines metabolic capacity of cells through the reduction of the tetrazolium salt (MTS), forming a brown formazan salt that is measured spectrophotometrically at 490 nm.

### Protein extraction

At 24 h post-injury, cells were lysed with 20 μL/well of RIPA buffer (mM: 150 NaCl, 5 EDTA, 50 Tris; %: 1.0 NP-40, 0.5 sodium deoxycholate, 0.1 SDS; pH 8.0) containing protease and phosphatase inhibitor cocktail (Roche Applied Science, Australia). Cell lysates from 8 wells within the same plate were pooled, and this was repeated four times with independent neuronal cultures. Lysates were clarified by centrifugation at 14,000 g for 5 min at 4 °C, and protein concentration determined via Bradford’s assay (Bio-Rad). Aliquots (3.5 mg/mL) of each treatment group were prepared for subsequent iTRAQ analysis and stored at − 20 °C prior to use.

### Protein sample preparation and iTRAQ labelling

Quantitative 4-plex iTRAQ proteomics analysis was performed on four independent protein samples for each treatment. Protein sample preparation and iTRAQ labeling was as previously described [25]. Briefly, the protein samples were de-salted, reduced, alkylated, and trypsin-digested according to the iTRAQ protocol [Sciex]. The resulting peptide samples were labeled with iTRAQ reagents as follows: 114: Untreated control (Cont); 115: glutamic acid treated (Glut); 116: R18 treated (R18); 117: R18 and glutamic acid treated (R18 + Glut). All labeled samples were combined to make a pooled sample. Peptides were desalted on a Strata-X 33 μM polymeric reversed phase column (Phenomenex) and dissolved in a buffer containing 2% acetonitrile 0.1% formic acid before separation by High pH on an Agilent 1100 HPLC system using a Zorbax C18 column (2.1 × 150 mm). Peptides were eluted with a linear gradient of 20 mM ammonium formate, 2% ACN to 20 mM ammonium formate, 90% ACN at 0.2 mL/min. The 95 fractions were concatenated into 12 fractions and dried down. Each fraction was analyzed by electrospray ionization mass spectrometry using the Shimadzu Prominence nano HPLC system [Shimadzu] coupled to a 5600 TripleTOF mass spectrometer [Sciex]. Samples were loaded onto an Agilent Zorbax 300SB-C18, 3.5 μm [Agilent Technologies] and separated with a linear gradient of water/acetonitrile/0.1% formic acid (v/v). Fourteen percent of the labeled sample was loaded on the mass spectrometer.

### Proteomic data analysis: qualification and quantitation

Spectral data was qualified using ProteinPilot™ 5.0 software [Sciex] against the SwissProt database, utilizing the *Rattus norvegicus* (Rat) taxonomy (Version: April 2017, 7,985 sequences; https://www.uniprot.org/proteomes/UP000002494). The False Discovery Rate (FDR) was automatically calculated by the Proteomics System Performance Evaluation Pipeline (PSPEP) feature in the ProteinPilot™ software (AB Sciex, Foster, CA, USA; Version 5.0.1) using the reversed version of the protein sequences contained in the search database. For quantitative protein analysis, a fold change in protein expression > ±1.3-fold with a *p* < 0.05 was considered to be a ‘differentially expressed protein’ (DEP). Protein expression changes with R18, Glut, and R18 + Glut treatment were compared to the control (Cont). In addition, protein changes with Glut treatment were compared with R18 + Glut treatment using Cont protein expression as baseline.

### Proteomic data analysis: bioinformatics

Gene ontology analysis with the ‘Protein ANalysis THrough Evolutionary Relationships’ (PANTHER; Version 14.0, released 2018-12-03; http://pantherdb.org/) classification system was utilized to categorize the collective DEPs in the R18, Glut, or R18 + Glut samples, relative to Cont sample, as well as Glut sample, relative to the Glut + R18 sample. These proteins were functionally categorized according to the domains of ‘biological processes’, ‘molecular functions’, and ‘cellular components’ [26].

Protein-protein interaction networks were identified using STRING (Version 11.0, released 2017-05-14; http://www.string-db.org/). STRING is a database of known and predicted physical and functional protein-protein interaction, generated through computational prediction from five key databases: ‘Genomic Context Predictions’, ‘High-throughput Lab Experiments’, ‘(Conserved) Co-Expression’, ‘Automated Textmining’, and ‘Previous Knowledge in Databases’. Cytoscape (Version 3.7.1) was subsequently utilized to construct and analyze the protein-protein interaction networks, and ‘Cluster with overlapping Neighbourhood Expansion’ (Cluster ONE; Version 14) was used for network clustering of protein-protein interactions, to identify densely connected and overlapping protein networks.

Identified DEPs were also imported into the Kyoto Encyclopedia of Genes and Genomes (KEGG) Pathway database (http://www.genome.jp/kegg/pathway.html) for analysis of common biological pathways and diseases associated with the DEPs.

### Statistical analysis

Statistical analysis was conducted with the Prism 8.0 GraphPad statistical software package. Cell viability data were expressed as mean ± S.E.M. of biological replicates, and multiple comparisons were conducted with one-way ANOVA and Bonferroni’s post hoc test to assess significance, with significance taken as *p* < 0.05. For Cytoscape network analysis, network cut-offs of > 3 proteins were utilized, and a one-sided Mann-Whitney U test was used to identify significant common networks (*p* < 0.05).

## Results

### R18 improves cell viability in uninjured and glutamic acid-treated neuronal cells

**Fig. 2:**
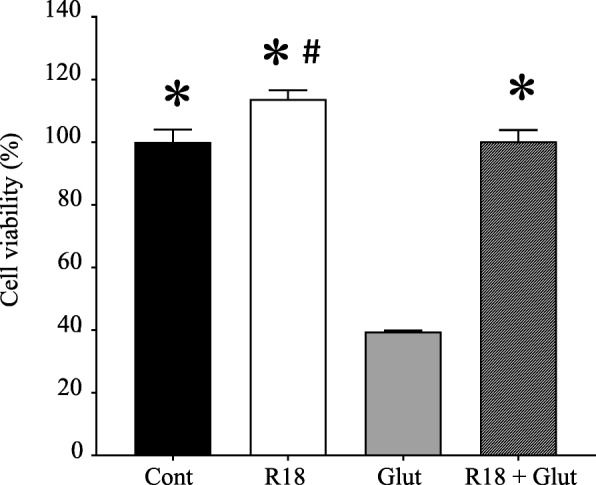
R18 provides potent neuroprotection against glutamic acid excitotoxicity in primary cortical neurons. Neuronal cultures were subjected to a 10-min R18 pre-treatment (2 μM) and subsequent 5-min glutamic acid exposure (Glut; 100 μM). MTS cell viability was assessed at 24 h post-injury. Cell viability was expressed as mean ± S.E.M (*p* < 0.05 relative to *Glut or #Cont)

In line with previous studies [12], R18 exhibited potent neuroprotection against glutamic excitotoxic injury in cortical neuronal cultures. In addition, as has been previously reported, cell viability was also significantly increased in neuronal cultures treated with R18 compared to control cultures (Fig. 2).

### Quantitative and qualitative proteomic analysis

iTRAQ proteomic analysis detected 7,528 distinct peptide fragments with > 95% confidence, resulting in the identification of 800 proteins (minimum of ≥2 matching peptide hits with > 95% confidence) consisting of a total of 140 distinct proteins (Table 1 and Additional file 1: Table S1). When compared to Cont, R18, Glut, and Glut + R18 differentially regulated 5, 95 and 14 proteins, respectively (Table 1; see Additional file 2: Table S2 for Glut + R18 DEPs). When compared to Glut, R18 + Glut differentially regulated 98 proteins (Table 1 and Additional file 2: Table S2).

**Table 1:** Differentially expressed proteins identified in neurons treated with: R18 (R18), glutamic acid (Glut), or R18 and glutamic acid exposure (R18 + Glut)

Gene name	SwissProt Accession Number	Protein	Fold up−/down-regulated^a^		
			R18 vs Cont	Glut vs Cont	R18 + Glut vs Glut
Mitochondrial respiration/function					
Acly	P16638	ATP-citrate synthase	1.076	**−2.399** ^b^	1.659
Aco2	Q9ER34	Aconitate hydratase, mitochondrial	−1.028	**−3.435**	**4.285**
Atp5a1	P15999	ATP synthase subunit alpha, mitochondrial	−1.472	**1.888**	**−2.421**
Atp5b	P10719	ATP synthase subunit beta, mitochondrial	−1.117	**2.558**	**−3.342**
Atp5h	P31399	ATP synthase subunit d, mitochondrial	1.076	**2.754** ^b^	−1.836
Atp5o	Q06647	ATP synthase subunit O, mitochondrial	1.406	**2.032** ^b^	−1.644
Cat	P04762	Catalase	−1.555	1.138	**−1.659** ^b^
Cox4i1	P10888	Cytochrome c oxidase subunit 4 isoform 1, mitochondrial	−1.259	**2.070**	**−2.704**
Idh1	P41562	Isocitrate dehydrogenase [NADP] cytoplasmic	1.066	**1.459**	**−1.486**
Mdh2	P04636	Malate dehydrogenase, mitochondrial	−1.247	**2.355**	**−2.535**
Ndufs1	Q66HF1	NADH-ubiquinone oxidoreductase 75 kDa subunit, mitochondrial	1.294	**2.443**	**−5.249**
Uqcrc2	P32551	Cytochrome b-c1 complex subunit 2, mitochondrial	−1.247	**1.888**	**−2.704**
Proteasome & Protein synthesis					
Asns	P49088	Asparagine synthetase [glutamine-hydrolyzing]	2.089	2.228	**2.421** ^b^
Cct2	Q5XIM9	T-complex protein 1 subunit beta	1.106	**−3.802**	**3.631**
Cct3	Q6P502	T-complex protein 1 subunit gamma	−1.158	**−4.787**	**4.207**
Cct4	Q7TPB1	T-complex protein 1 subunit delta	1.127	**−3.944**	**3.499**
Cct5	Q68FQ0	T-complex protein 1 subunit epsilon	1.096	**−2.269**	**2.291**
Psmc1	P62193	26S protease regulatory subunit 4	−1.419	**−22.67**	**18.197**
Axonal growth/Neuronal differentiation/Cytoskeletal arrangement					
Actr2	Q5M7U6	Actin-related protein 2	−1.355	− 1.271	**− 1.459** ^b^
Ap2m1	P84092	AP-2 complex subunit mu	1.271	**−5.394**	**6.855**
Armc10	B1WBW4	Armadillo repeat-containing protein 10	−1.294	**−6.667**	**2.070**
Baiap2	Q6GMN2	Brain-specific angiogenesis inhibitor 1-associated protein 2	−1.331	**−90.09**	**99.083**
Basp1	Q05175	Brain acid soluble protein 1	**1.343**	**1.614**	**−1.270**
Cntn1	Q63198	Contactin-1	−1.117	**1.977**	**−1.836**
Cttn	Q66HL2	Src substrate cortactin	1.117	**−5.345** ^b^	3.251
Dcx	Q9ESI7	Neuronal migration protein doublecortin	1.000	**−6.139**	**6.026**
Dnm1	P21575	Dynamin-1	−2.148	**−4.093**	**3.076**
Dpysl2	P47942	Dihydropyrimidinase-related protein 2	−1.225	**1.723**	**−1.690**
Dync1h1	P38650	Cytoplasmic dynein 1 heavy chain 1	−1.087	**−8.873**	**8.472**
Fabp7	P55051	Fatty acid-binding protein, brain	−1.028	**4.406** ^b^	−4.207
Fyn	Q62844	Tyrosine-protein kinase Fyn	1.117	−2.489	**2.754** ^b^
Gdi1	P50398	Rab GDP dissociation inhibitor alpha	−1.419	**1.820**	**−2.965**
Gfap	P47819	Glial fibrillary acidic protein	1.4322	**1.906** ^b^	−1.159
Kif21b	F1M5N7	Kinesin-like protein KIF21B	−1.282	**−17.857**	**16.444**
Krt1	Q6IMF3	Keratin, type II cytoskeletal	**−4.055** ^b^	−1.236	−6.485
Krt10	Q6IFW6	Keratin, type I cytoskeletal 10	−2.168	−1.722	**−16.892** ^b^
Map2	P15146	Microtubule-associated protein 2	1.138	**−8.628**	**11.482**
Map4	Q5M7W5	Microtubule-associated protein 4	−2.884	**−4.488**	**4.169**
Mapt	P19332	Microtubule-associated protein tau	1.077	**−7.179**	**7.379**
Myh10	Q9JLT0	Myosin-10	−1.459	**−2.466**	**2.228**
Ncam1	P13596	Neural cell adhesion molecule 1	−1.180	**2.831**	**−4.093**
Pa2 g4	Q6AYD3	Proliferation-associated protein 2G4	1.202	−2.188	**2.489** ^b^
Pak3	Q62829	Serine/threonine-protein kinase PAK 3	−2.208	−4.365	**5.754** ^b^
^b^Pebp1	P31044	Phosphatidylethanolamine-binding protein 1	1.5276	**2.780** ^b^	−2.128
Rala	P63322	Ras-related protein Ral-A	−1.236	**2.704** ^b^	−1.690
Rtn4	Q9JK11	Reticulon-4	1.107	**−2.148**	**2.679**
Tpm4	P09495	Tropomyosin alpha-4 chain	−1.097	−2.270	**1.738** ^b^
Tuba4a	Q5XIF6	Tubulin alpha-4A chain	1.086	**−2.679** ^b^	2.535
Tubb5	P69897	Tubulin beta-5 chain	−1.038	**−18.018**	**16.444**
Vesicular/Transmembrane trafficking					
Actn4	Q9QXQ0	Alpha-actinin-4	−1.294	**1.906** ^b^	−1.381
Actr1a	P85515	Alpha-centractin	−1.738	**−3.565** ^b^	2.148
Ap2a2	P18484	AP-2 complex subunit alpha-2	1.159	−2.582	**3.698** ^b^
Atp1a3	P06687	Sodium/potassium-transporting ATPase subunit alpha-3	**−2.128**	**1.660**	**−2.355**
Cadps	Q62717	Calcium-dependent secretion activator 1	−1.486	**−11.481**	**6.194**
Camk2a	P11275	Calcium/calmodulin-dependent protein kinase type II subunit alpha	−1.419	**−6.667**	**6.918**
Cask	Q62915	Peripheral plasma membrane protein CASK	1.514	1.306	**−1.888** ^b^
Dpysl5	Q9JHU0	Dihydropyrimidinase-related protein 5	−1.180	1.213	**−1.459** ^b^
Klc1	P37285	Kinesin light chain 1	1.107	**−6.024** ^b^	5.058
Nsf	Q9QUL6	Vesicle-fusing ATPase	−1.117	**−5.807**	**4.656**
Prkar2b	P12369	cAMP-dependent protein kinase type II-beta regulatory subunit	−1.472	**−3.105**	**1.754**
Stx1b	P61265	Syntaxin-1B	1.225	**1.600**	**−1.419**
Stxbp1	P61765	Syntaxin-binding protein 1	−1.107	**1.600**	**−2.466**
Syn1	P09951	Synapsin-1	−1.786	**−3.597** ^b^	3.3113
ER proteostasis/Protein modification					
Calr	P18418	Calreticulin	1.419	**1.871**	**−1.570**
Ddost	Q641Y0	Oligosaccharyl transferase 48 kDa subunit	−1.500	**2.466**	**−2.938**
Erp29	P52555	Endoplasmic reticulum resident protein 29	−1.067	**2.168** ^b^	−3.163
Hsp90aa1	P82995	Heat shock protein HSP 90-alpha	1.038	1.282	**−1.446** ^b^
Hspa5	P06761	78 kDa glucose-regulated protein	1.038	**2.014**	**−1.542**
Hspa8	P63018	Heat shock cognate 71 kDa protein	1.259	−2.208	**2.559** ^b^
Hspa9	P48721	Stress-70 protein, mitochondrial	1.159	**−6.083**	**8.318**
Hspd1	P63039	60 kDa heat shock protein, mitochondrial	1.107	**1.486**	**−1.556**
Pdia3	P11598	Protein disulfide-isomerase A3	−1.600	**1.614**	**−1.995**
Phb	P67779	Prohibitin	−1.213	**1.644**	**−2.535**
Phb2	Q5XIH7	Prohibitin-2	−1.047	**3.837**	**−2.992**
Por	P00388	NADPH--cytochrome P450 reductase	−1.107	−1.038	**1.343** ^b^
Tcp1	P28480	T-complex protein 1 subunit alpha	−1.472	**−3.597** ^b^	2.911
Uba1	Q5U300	Ubiquitin-like modifier-activating enzyme 1	−1.107	**−5.105**	**4.406**
Glycolysis & Carbohydrate metabolism					
Alb	P02770	Serum albumin	−1.570	**1.722**	**−5.701**
Aldoa	P05065	Fructose-bisphosphate aldolase A	−1.660	**1.629**	**−2.355**
Eno1	P04764	Alpha-enolase	1.107	**2.911**	**−1.706**
Gapdh	P04797	Glyceraldehyde-3-phosphate dehydrogenase	−1.028	**−11.700**	**10.280**
Gpi	Q6P6V0	Glucose-6-phosphate isomerase	−1.318	**1.754**	**−3.435**
Hk1	P05708	Hexokinase-1	1.138	**2.051** ^b^	−1.888
Ldha	P04642	L-lactate dehydrogenase A chain	−1.786	1.028	**−1.675** ^b^
Pkm	P11980	Pyruvate kinase PKM	−1.009	**1.787**	**−1.941**
Taldo1	Q9EQS0	Transaldolase	1.259	**−1.837**	**2.109**
Mitochondrial fatty acid synthesis					
Acat1	P17764	Acetyl-CoA acetyltransferase, mitochondrial	1.159	**2.377**	**−1.500**
Bdh1	P29147	D-beta-hydroxybutyrate dehydrogenase, mitochondrial	−1.213	**−5.970**	**4.246**
Fasn	P12785	Fatty acid synthase	−1.117	**−3.311**	**2.704**
Got2	P00507	Aspartate aminotransferase, mitochondrial	−1.923	1.213	**−2.109** ^b^
Ribosome components/RNA trafficking & processing					
Aars	P50475	Alanine--tRNA ligase, cytoplasmic	−1.556	**−5.495**	**4.699**
C1qbp	O35796	ASF/SF2-associated protein p32	1.213	**1.600** ^b^	−1.888
Ddx1	Q641Y8	ATP-dependent RNA helicase DDX1	−1.148	**−11.173**	**4.286**
Eef1a1	P62630	Elongation factor 1-alpha 1	−1.225	**−5.444**	**4.529**
Eef2	P05197	Elongation factor 2	−1.202	**−4.131**	**3.048**
Eif4a2	Q5RKI1	Eukaryotic initiation factor 4A-II	−1.057	**−3.945**	**3.02**
Eif5a	Q3T1J1	Eukaryotic translation initiation factor 5A-1	1.514	**−6.983**	**9.638**
Elavl2	Q8CH84	ELAV-like protein 2	−1.107	−4.405	**5.598** ^b^
Hnrnpa1	P04256	Heterogeneous nuclear ribonucleoprotein A1	−1.076	**−9.911**	**9.462**
Hnrnpa2b1	A7VJC2	Heterogeneous nuclear ribonucleoproteins A2/B1	1.318	**−4.325**	**6.138**
Hnrnpa3	Q6URK4	Heterogeneous nuclear ribonucleoprotein A3	1.191	**−3.311**	**4.920**
Hnrnpd	Q9JJ54	Heterogeneous nuclear ribonucleoprotein D0	1.202	**−5.495**	**6.252**
Hnrnpk	P61980	Heterogeneous nuclear ribonucleoprotein K	−1.057	**−8.091**	**8.872**
Hnrnpl	F1LQ48	Heterogeneous nuclear ribonucleoprotein L	−1.028	−2.729	**4.207** ^b^
Khsrp	Q99PF5	Far upstream element-binding protein 2	1.556	−3.908	**8.017** ^b^
Matr3	P43244	Matrin-3	−1.500	**−3.373** ^b^	3.945
Rpl7	P05426	60S ribosomal protein L7	1.225	−2.109	**2.188** ^b^
Rpl13	P41123	60S ribosomal protein L13	−1.057	−2.377	**2.754** ^b^
Rplp0	P19945	60S acidic ribosomal protein P0	**1.486** ^b^	−1.191	−1.076
Rps24	P62850	40S ribosomal protein S24	1.854	−5.754	**14.06** ^b^
Rps27	Q71TY3	40S ribosomal protein S27	−1.102	**−18.18** ^b^	5.058
Yars	Q4KM49	Tyrosine--tRNA ligase, cytoplasmic	−1.225	**−4.656**	**3.342**
Calcium transport and signalling					
Vdac1	Q9Z2L0	Voltage-dependent anion-selective channel protein 1	−1.419	**2.466**	**−2.938**
Cacna2d1	P54290	Voltage-dependent calcium channel subunit alpha-2/delta-1	1.306	**4.207**	**−4.018**
Gnao1	P59215	Guanine nucleotide-binding protein G(o) subunit alpha	−1.148	**−5.152**	**3.802**
Letm1	Q5XIN6	LETM1 and EF-hand domain-containing protein 1, mitochondrial	−3.020	**1.459** ^b^	−1.236
Nudt3	Q566C7	Diphosphoinositol polyphosphate phosphohydrolase 1	**1.459** ^b^	1.282	1.117
Ywhaq	P68255	14–3-3 protein theta	−1.087	**1.355**	**−1.615**
Miscellaneous					
Ak1	P39069	Adenylate kinase isoenzyme 1 (*Cellular energy homeostasis*)	1.009	**−2.938**	**4.325**
Atic	O35567	Bifunctional purine biosynthesis protein PURH (*Purine biosynthesis*)	1.038	1.923	**−2.630** ^b^
Dnm1l	O35303	Dynamin-1-like protein (*Mitochondrial fission*)	−1.820	**−7.519** ^b^	5.297
Hist1h4b	P62804	Histone H4 (*Transcription regulation*)	1.754	−3.802	**7.516** ^b^

^a^ Statistically significant values (*p* < 0.05) for fold up−/down-regulation ≥1.3-fold are highlighted in bold. ^b^ 25 uniquely DEPs in R18 and Glut treatment versus Cont, or R18 + Glut treatment versus Glut

#### Proteins regulated by R18 treatment alone (R18 vs Cont)

Of the five DEPs identified in the R18 sample, three were uniquely regulated (Table 1). Two of these proteins were upregulated: 60S acidic ribosomal protein P0 (Rplp0; 1.49) and Diphosphoinositol polyphosphate phosphohydrolase 1 (Nudt3; 1.46), and one was downregulated: keratin, type II cytoskeletal (Krt1; − 4.05).

#### Proteins regulated by glutamic acid injury alone (glut vs Cont)

Of the 95 DEPs identified in the Glut sample, 21 were uniquely regulated (Table 1). The greatest magnitude fold-change in a down-regulated protein was observed for brain-specific angiogenesis inhibitor 1-associated protein 2 (Baiap2; − 90.09), while the greatest fold-change in an up-regulated protein was for fatty acid-binding protein, brain (Fabp7; 4.41).

#### Proteins regulated by R18 plus glutamic acid injury (R18 + Glut vs Cont)

Of the 14 DEPs identified in the R18 + Glut sample none were uniquely regulated (Additional file 2: Table S2). The greatest magnitude fold-change in an up-regulated protein was observed for UV excision repair protein RAD23 homolog B (Rad23b; 3.251), while the greatest fold-change in a down-regulated protein was for Keratin, type I cytoskeletal 10 (Krt10; − 33.333).

#### Proteins regulated by R18 plus glutamic acid injury vs glutamic acid injury alone (R18 + Glut vs glut)

**Fig. 3:**
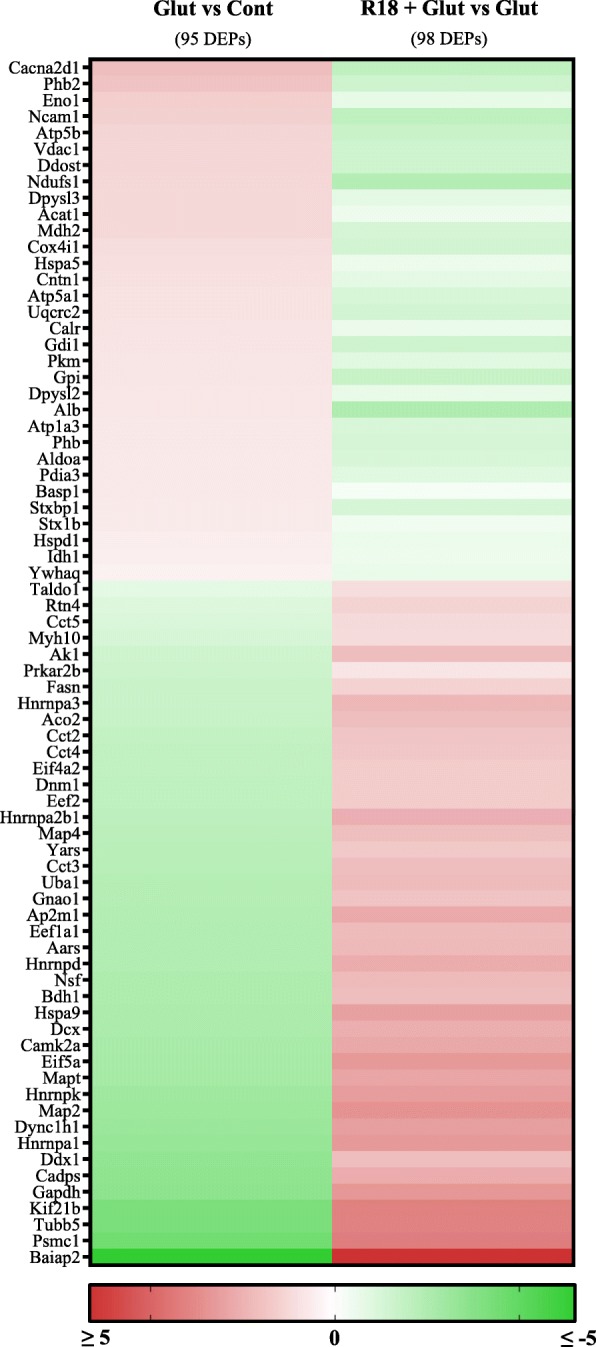
Heatmap of differentially expressed proteins (DEPs) regulated by R18 vs Cont, Glut vs Cont, or R18 + Glut vs Glut. Protein expression changes are shown as log(2) of fold-change

Of the 98 DEPs identified after R18 + Glut treatment (R18 + Glut vs Glut), 73 of the proteins were also regulated by R18 and/or Glut treatments alone, and 25 were uniquely regulated (Table 1; uniquely regulated proteins indicated by *). In addition, R18 treatment reversed the up- or down-regulation of all 73 DEPs (Table 1, Fig. 3). Of the 25 uniquely regulated proteins, the greatest magnitude fold-change in protein expression observed was with Brain-specific angiogenesis inhibitor 1-associated protein 2 (Baiap2; − 99.08).

### Functional categorization of differentially regulated proteins (DEPs)

**Fig. 4:**
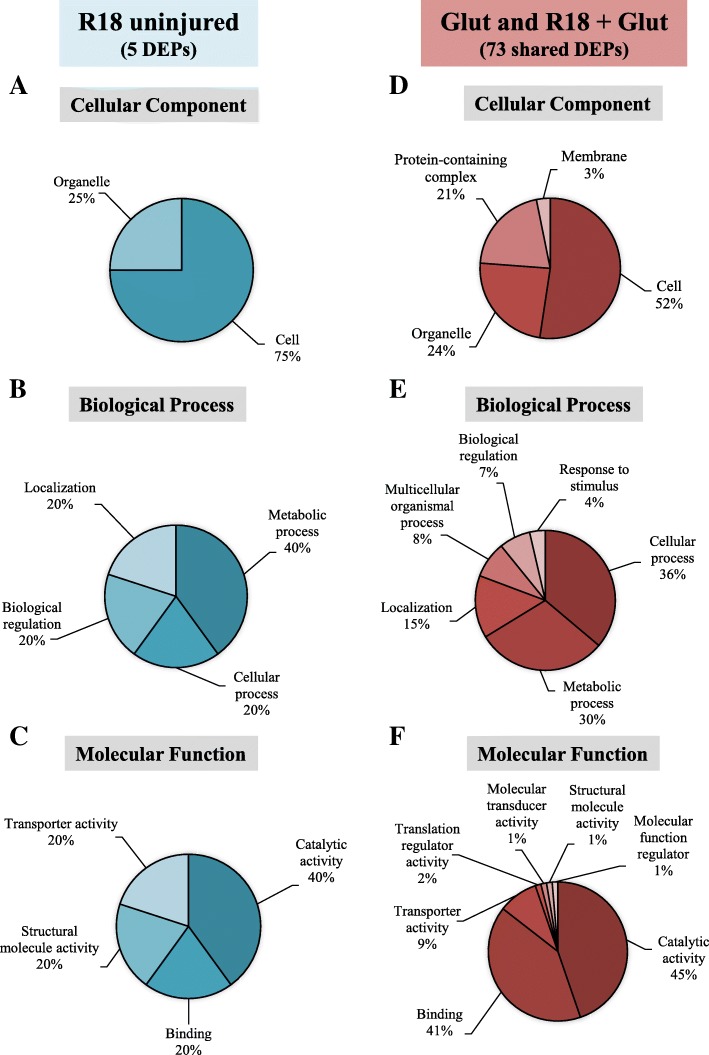
PANTHER gene-ontology functional categorization of DEPs regulated by R18 in healthy neurons (vs Cont; blue), and R18 + Glut in injured neurons (vs Glut; red). The top five categories are displayed across the functional categories of **(a, b)** Molecular Function **(c, d)** Biological Process, and **(e, f)** Cellular Component

For further functional characterization of DEPs we focused on protein changes in the R18 and R18 + Glut treatments groups as we were most interested in the effects of R18 on proteins regulated in uninjured and injured neurons. PANTHER Gene Ontology analysis was utilized to categorize the DEPs regulated by R18 alone (5 proteins; R18 vs Cont), and the DEPs regulated by R18 + Glut treatment (73 proteins; R18 + Glut vs Glut) according to ‘cellular component’ (Fig. 4a and d) ‘biological process’ (Fig. 4b and e), and ‘molecular function’ (Fig. 4c and f) (Full data provided in Additional file 3: Table S3).

**Fig. 5:**
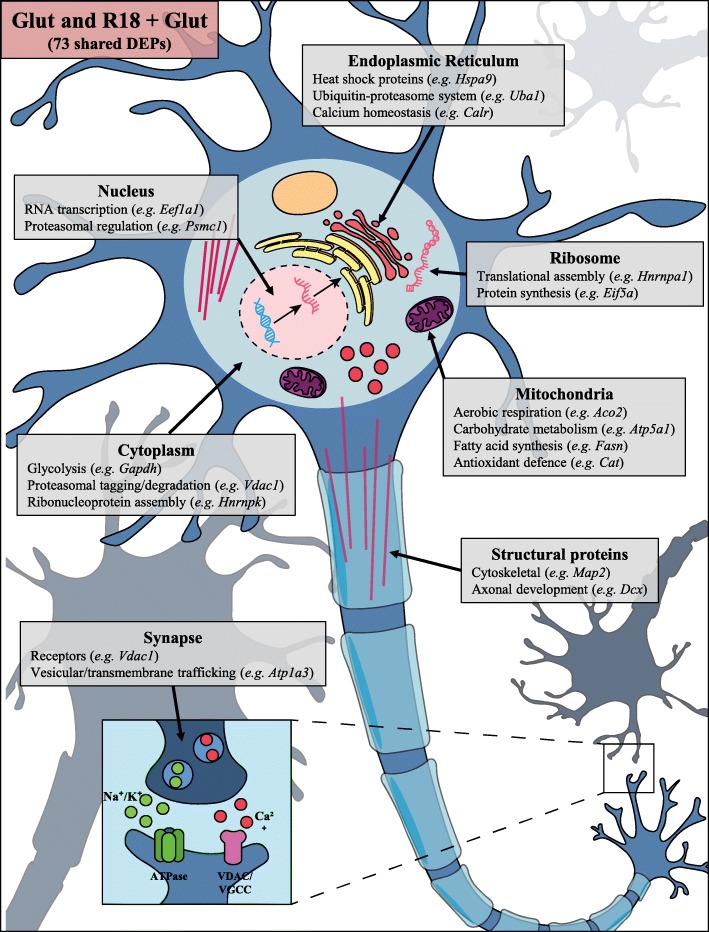
Schematic representation of representative shared neuronal proteins regulated by glutamic acid excitotoxicity (Glut) and R18 + Glut, based on location and function. Comprehensive protein changes are detailed in Table 1

The 5 DEPs regulated by R18 treatment included proteins located in the nucleus and ribosomes which catalyze purine nucleotide catabolic activity (e.g. Nudt3), and modulate rRNA binding (e.g. Rplp0). Other proteins regulated by R18 are involved in intracellular ion trafficking (e.g. Atp1a3) and cytoskeletal structure (e.g. Krt1) (Table 1).

The 73 DEPs regulated by R18 + Glut treatment included proteins involved in mitochondrial respiration and function (e.g. Aco2 and Atp5a1), proteasomal regulation and protein synthesis (e.g. Psmc1 and Cct3), proteostasis/protein modification in the endoplasmic reticulum (e.g. Hspa9 and Uba1), and RNA trafficking/processing (e.g. Eif5a and Hnrnpa1), as well as cytoskeletal rearrangement and axonal growth (e.g. Map2 and Dcx) and vesicular/membrane trafficking (e.g. Atp1a3 and Camk2a). Significant changes of key neuronal proteins are summarized in the schematic detailed in Fig. 5 (full data available in Additional file 2: Table S2 and Additional file 3: Table S3).

**Fig. 6:**
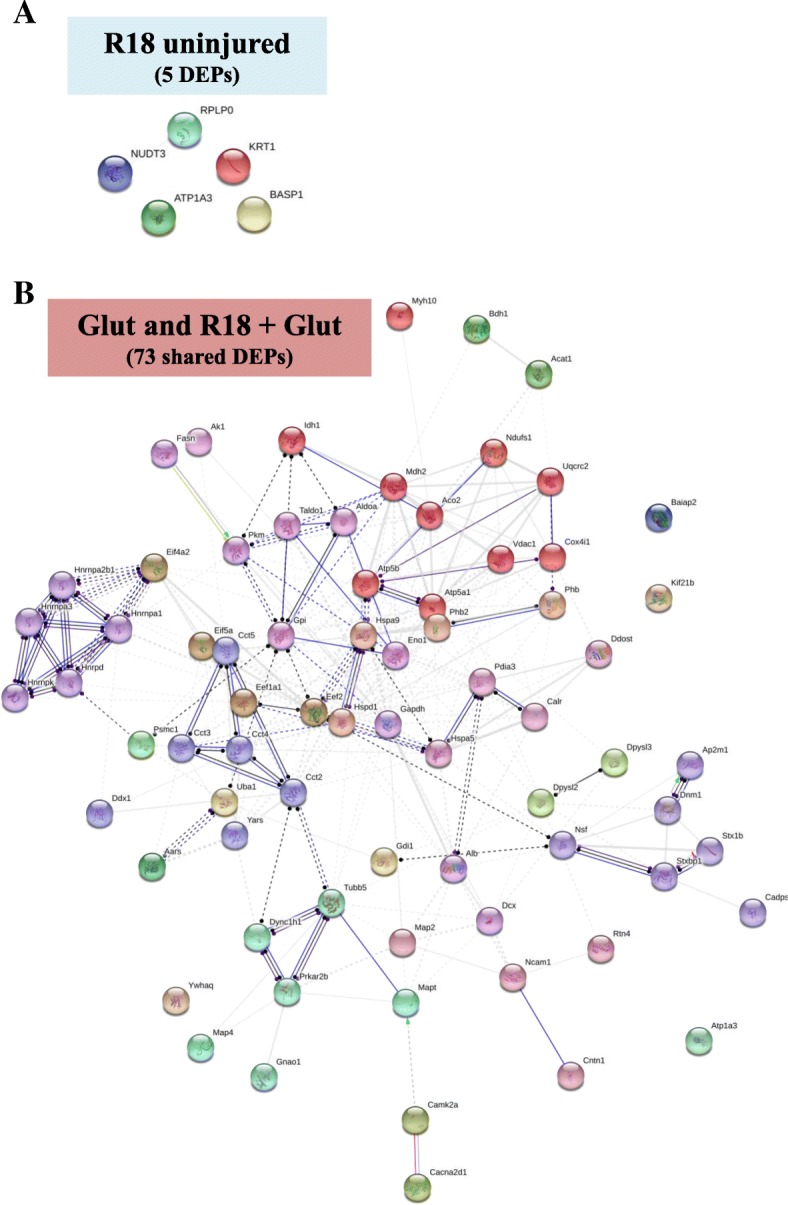
Enriched STRING PPI network analysis of DEPs regulated by **a.** R18 treatment in healthy neurons (vs Cont), and **b.** DEPs commonly regulated by Glut and R18 + Glut (vs Glut), demonstrating molecular actions of direct and indirect protein-protein interactions between significantly regulated proteins. STRING parameters were set to high confidence (0.700), with only query proteins shown

### Protein-protein interaction network analysis of DEPs regulated by R18 in uninjured and glutamic acid injured neurons

STRING analysis was used to identify potential protein-protein interactions across the 5 DEPs regulated by R18 treatment, and the 73 DEPs regulated by Glut and R18 + Glut. No significant protein-protein interactions were identified for the 5 DEPs regulated by R18 treatment (R18 vs Cont) (Fig. 6a). Two hundred and twenty-two nodes representing direct and indirect protein-protein interactions were identified for the 73 DEPs regulated by R18 + Glut treatment (R18 + Glut vs Glut) (Fig. 6b).

**Fig. 7:**
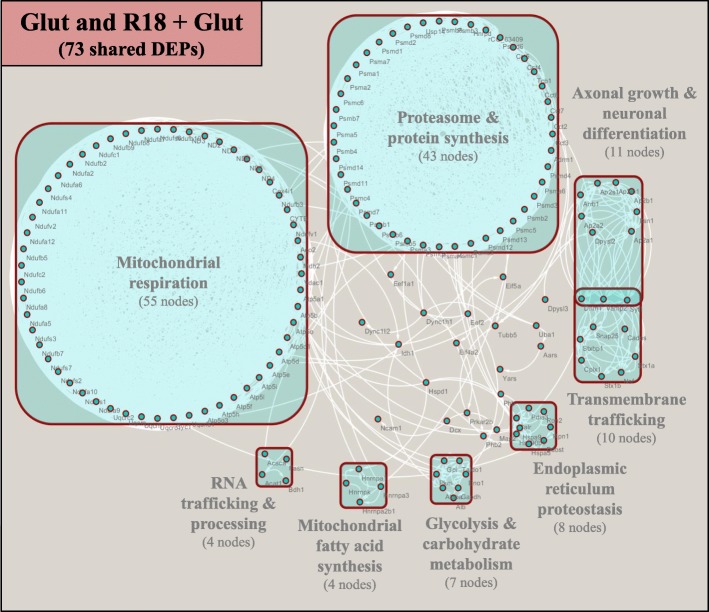
Cytoscape ClusterONE analysis of enriched STRING protein-protein interaction network of shared DEPs regulated by both Glut and R18 + Glut. Cytoscape ClusterONE analysis was used to group protein clusters based on their involvement in ‘Mitochondrial respiration’, ‘Axonal growth and neuronal differentiation’, ‘Transmembrane trafficking’, ‘Endoplasmic reticulum proteostasis’, ‘Glycolysis and carbohydrate metabolism’, ‘Mitochondrial fatty acid synthesis’, or ‘RNA trafficking and processing’. Clusters represent statistical significance cut-offs of *p* < 0.05, and empty nodes represent proteins that do not share statistically significant functions with other proteins

ClusterONE network analysis of the 222 nodes revealed that the protein-protein interactions could be grouped into eight clusters representing distinct biological functional entities (Fig. 7; boxed proteins). The clusters were classified as ‘Mitochondrial respiration’ (55 nodes), ‘Proteasome and Protein synthesis’ (43 nodes), ‘Axonal growth & neuronal differentiation’ (11 nodes), ‘Transmembrane trafficking’ (10 nodes), ‘Endoplasmic reticulum proteostasis’ (8 nodes), ‘Glycolysis and carbohydrate metabolism’ (7 nodes), ‘RNA trafficking and processing’ (4 nodes), and ‘Mitochondrial fatty acid synthesis’ (4 nodes) (Full data provided in Additional file 4: Table S4).

### KEGG pathway analysis of 73 shared proteins

**Fig. 8:**
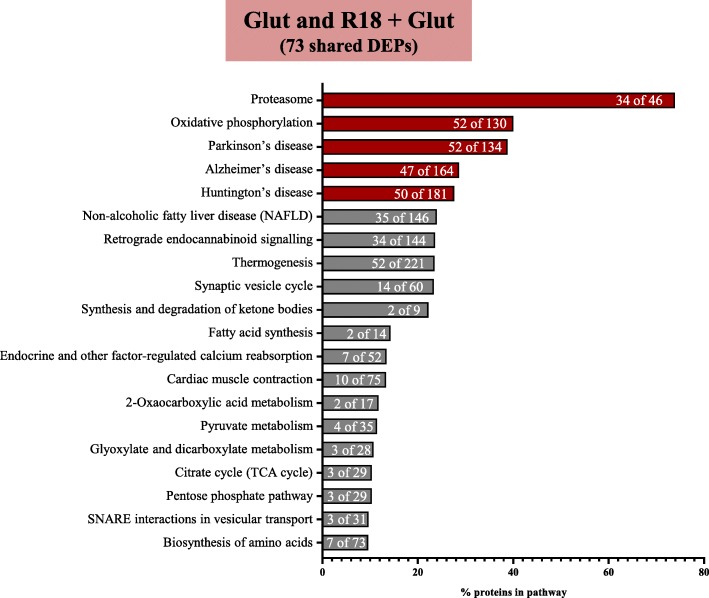
Enriched KEGG pathway analysis of shared DEPs common to both Glut and R18 + Glut (vs Glut), demonstrating the top 20 pathways. Shown above is the percentage of proteins mapped to the respective significantly regulated pathways, with the fraction of regulated proteins mapping onto the total number of proteins in each pathway provided in white. The top 5 pathways are outlined in red

KEGG pathway analysis to determine the biological pathways and diseases associated with 222 protein-protein interactions identified pathways pertaining to proteostasis (‘Proteasome’; 34 of 46 proteins), energy metabolism (‘Oxidative phosphorylation’; 52 of 130 proteins), and neurotransmission (‘Synaptic vesicle cycle’; 14 of 60 proteins), and ‘Retrograde endocannabinoid signaling’; 34 of 144 proteins) (Fig. 8; full data in Additional file 5: Table S5). In addition, KEGG analysis revealed that the protein-protein interactions were associated with the neurodegenerative disorders Parkinson’s disease (PD; 52 of 134 proteins), Alzheimer’s disease (AD; 47 of 164 proteins), and Huntington’s disease (HD; 50 of 181 proteins).

## Discussion

In recent years, CARPs have emerged as a novel class of potential neuroprotective therapeutics for a broad range of acute brain injuries and chronic neurodegenerative disorders. These CARPs include short-chained poly-arginine peptides [11, 13, 27], SS-peptides [28, 29], APOE-derived peptides [27, 30], and TAT-fused peptides, including TAT-NR2B9c (NA-1) and JNK1-TAT [11, 31]. Such CARPs have been shown to exert their neuroprotective action through a variety of targets, which include structural and functional preservation of mitochondria [32], reduced ROS generation [33], inhibition of protein aggregation [34], modulation of glutamate or calcium ion receptors (excitotoxicity/calcium influx) [35], and activation of pro-survival signaling [36, 37]. Given the diverse biochemical and cellular effects CARPs can exert on cells, it is likely that other cytoprotective processes are also involved, which have yet to be fully elucidated. To this end, the present study is the first to employ an iTRAQ proteomics approach to gain insight into protein expression changes after poly-arginine-18 (R18) treatment of uninjured neuronal cultures and neuronal cultures subjected to glutamic acid excitotoxic injury.

### The effects of R18 treatment on uninjured neurons

Proteomics analysis of neuronal cultures 24-h after a 10-min exposure to R18 identified 5 DEPs. The small number of detected protein expression changes was surprising given that CARPs can induce a variety of biological effects on cells [38]. However, it is likely that the small number of proteins detected was in part due to the 24-h post-R18 treatment time point used to analyze protein expression changes, as majority of the protein changes elicited by R18 treatment may potentially occur within the first few hours, and as such, may no longer have been detectable or did not fit the requirements for classification of a DEP (e.g. ± > 1.3 fold change) by 24 h.

The proteins that were identified as being affected by R18 were largely associated with protein synthesis and transmembrane protein and cationic ion transport, and did not significantly map onto KEGG pathways, suggesting that the R18 peptide does not exert long-term biological effects in uninjured neurons. This is in line with the proposed notion that neuroprotective agents should preferentially interact with and/or modulate cellular targets activated following pathological events to minimize the chance of off-target side effects. Such protective agents are deemed ‘pathologically-activated’ therapeutics, which are thought to have a particularly useful application in neurological disorders, as brain tissue is especially susceptible to drug-induced disruptions and unwanted drug side-effects [38, 39]. However, to provide a more comprehensive assessment of the biological effects of R18 on uninjured neurons, future studies should examine protein expression at earlier time points.

### R18 treatment reduces glutamic acid-induced changes in protein expression

An important finding of this study was the ability of R18 treatment to reverse the majority (74.5%; 73 of 98) of the protein changes induced by glutamic acid excitotoxicity, and thereby preserve the protein expression profiles of cortical neurons post-insult (Table 1; Additional file 4: Table S4). Further analysis revealed that these protein changes underpin key cellular functions, such as mitochondrial respiration and energy production, proteostasis, neuronal transmembrane trafficking, and RNA processing, which are dysregulated by excitotoxicity. Moreover, KEGG analysis of protein-protein interactions indicated predominant involvement of pathways pertaining to the proteasome and oxidative phosphorylation, which also represent two central biological processes underpinning aspects of neurodegenerative pathophysiology. This likely contributed to the identification of enriched protein-protein interactions pertaining to Parkinson’s disease, Alzheimer’s disease and Huntington’s disease.

Severe and/or prolonged disruptions in the ubiquitin-proteasome system have been implicated in both acute (ischaemic stroke, TBI) and chronic (AD, PD, motor neuron disease) neurological disorders [39, 40]. Previous studies have shown that CARPs exhibit proteasomal modulatory activity and could potentially conserve protein expression profiles through inhibition of injury-induced proteasomal protein degradation. For example, the arginine-rich PR-11 (H-RRRPRPPYLPRPRPPPFFPPRLPPRIPPGFPPRFPPRFP-OH; net charge + 11) and PR-39 (H-RRRPRPPYLPR-OH; net charge + 5) peptides attenuate inflammation induced by ischaemia-reperfusion injury through inhibition of proteasomal degradation of IκBα; a NFκB inhibitory protein [41, 42]. Taken together, it appears CARPs can influence the function of the proteasome, and thereby exert neuroprotective benefits during times of cellular stress.

Proteomic analysis also revealed that R18 preserved protein expression profiles pertaining to mitochondrial bioenergetics and structural integrity. Mitochondria are central mediators of intracellular calcium signaling events during excitotoxicity, and as such, are considered the “judge, jury, and executioner” of the cell [31, 43]. During excitotoxic injury, mitochondria act as a buffer for toxic intracellular calcium accumulation, however excessive mitochondrial calcium uptake can disrupt their structural and functional integrity, resulting in the release of pro-death signaling proteins from the mitochondrial inter-membrane space [44, 45]. Therefore, the ability of R18 treatment to attenuate excitotoxicity-induced protein changes underlying loss of mitochondrial integrity, provides evidence that the peptide helps preserve the function of the organelle in times of cellular stress. In line with the ability of CARPs to maintain mitochondrial function and energy generation, in this and previous studies R18 was demonstrated to increase MTS metabolism in uninjured neurons and in neurons after exposure to glutamic acid [14].

Bioinformatic analysis of the DEPs identified in the present study largely focused on the 73 proteins up- or down-regulated by glutamic acid excitotoxic injury. However, it is important to note that 25 other DEPs were also identified to be uniquely regulated by combined R18 + Glut treatment, which may represent additional proteins influenced by R18 and associated with neuroprotection. Alternatively, these proteins could reflect non-specific changes in protein expression unrelated to neuroprotection.

### Limitations and future directions

The proteomics methodology used in this study does not provide insight into other forms of protein modification, such as post-translational changes (e.g. phosphorylation, acetylation, and glycosylation), which may influence protein functions important for neuroprotection. In addition, only a 24-h timepoint was examined and therefore it would also be of interest to examine protein expression changes, as well as post-translational modifications at earlier timepoints after R18 treatment. Further studies are also required to confirm if the DEPs and the biochemical and disease pathways influenced by R18 treatment after glutamic acid excitotoxicity in vitro are also affected by the peptide in animal models of acute brain injury (e.g. stroke, TBI) and chronic neurodegenerative disorders (AD, PD).

## Conclusion

This exploratory study has demonstrated for the first time that the poly-arginine peptide R18 exerts significant effects in attenuating the protein expression changes associated with neuronal excitotoxicity in vitro*,* while inducing minimal changes in uninjured neurons. Collectively, our findings indicate that the neuroprotective effects of R18 following excitotoxicity are associated predominantly with preservation of neuronal proteostasis, together with positive effects on mitochondrial and proteasomal function. The findings of this study provide further evidence supporting the role of poly-arginine peptides as a potential neuroprotective therapeutic for both acute and chronic neurodegenerative disorders.

## Additional files

Additional file 1: **Table S1.** Summary of LC-MS/MS spectral data analysis. Summary of LC-MS/MS spectral data analysis with ProteinPilot™ 5.0 Software [Sciex] using the SwissProt database (Version April 2017; 7,985 sequences) against *Rattus norvegicus* (Rat) taxonomy, using the reversed version of the protein sequences contained in the search database. FDR was automatically calculated with the Proteomics System Performance Evaluation Pipeline (PSPEP) feature in the ProteinPilot™ software. (DOCX 17 kb)

Additional file 2: **Table S2.** Full iTRAQ proteomics data. Full iTRAQ proteomics data showing relative fold changes in protein expression and corresponding *p*-values. (DOCX 54 kb)

Additional file 3: **Table S3.** PANTHER gene-ontology functional categorization. PANTHER gene-ontology functional categorization of DEPs significantly regulated by R18 treatment alone (R18 vs Cont), glutamic acid exposure (Glut vs Cont), and R18 pre-treatment with glutamic acid exposure (R18 + Glut vs Glut). Note: proteins may have multiple functions, and as such, the total number of proteins in each category may be greater than the sum of DEPs across each treatment group. (DOCX 49 kb)

Additional file 4: **Table S4.** Cytoscape quantitative analysis. Cytoscape quantitative analysis of STRING data cluster strength for 73 shared DEPs across Glut and R18 + Glut treatment groups. (DOCX 56 kb)

Additional file 5: **Table S5.** Quantitative data and full gene list of KEGG pathway analysis. Quantitative data and full gene list of KEGG pathway analysis of 73 shared DEPs across Glut and R18 + Glut treatment groups, with details provided on term ID, overserved gene count vs. background gene count, and FDR. (DOCX 17 kb)

## Data Availability

All data generated or analysed during this study are included in this published article (and its Suppl. information files).

## Abbreviations

AD: Alzheimer’s disease
ALS: Amyotrophic lateral sclerosis
Cont: ‘No treatment’ group
DEP: Differentially expressed proteins
Glut: ‘Glutamic acid excitotoxic injury’ treatment group
HD: Huntington’s disease
LC-MS/MS: Liquid chromatography-tandem mass spectrometry
PD: Parkinson’s disease
R18 + Glut: ‘R18-treatment and glutamic acid injury’ treatment group
R18: Poly-arginine-18 peptide
