# Supplementary material for: Proteomic analysis of cortical neuronal cultures treated with poly-arginine peptide-18 (R18) and exposed to glutamic acid excitotoxicity
Source: Mol Brain. 2019 Jul 17;12:66. doi: 10.1186/s13041-019-0486-8 (PMC6637488; doi:10.1186/s13041-019-0486-8)
Supplement: Supplementary file 1 — Table S1. Summary of LC-MS/MS spectral data analysis. Summary of LC-MS/MS spectral data analysis with ProteinPilot™ 5.0 Software [Sciex] using the SwissProt database (Version April 2017; 7,985 sequences) against Rattus norvegicus (Rat) taxonomy, using the reversed version of the protein sequences contained in the search database. FDR was automatically calculated with the Proteomics System Performance Evaluation Pipeline (PSPEP) feature in the ProteinPilot™ software. (DOCX 17 kb) [file 13041_2019_486_MOESM1_ESM.docx]

**Additional file 1: Table S1.** Summary of LC-MS/MS spectral data analysis with ProteinPilot™ 5.0 Software [Sciex] using the SwissProt database (Version April 2017; 7,985 sequences) against *Rattus norvegicus* (Rat) taxonomy, using the reversed version of the protein sequences contained in the search database. FDR was automatically calculated with the Proteomics System Performance Evaluation Pipeline (PSPEP) feature in the ProteinPilot™ software.

| **Features** | | **Value** |
| --- | --- | --- |
| Number of proteins detected (≥1 peptide with >95% confidence) | | 949 |
| Number of proteins detected (≥2 peptide with >95% confidence) | | 800 |
| Number of distinct peptides with >95% confidence | | 7528 |
| Normalization | Glut vs Cont | 0.5 |
|  | R18 vs Cont | 1 |
|  | R18 + Glut vs Cont | 1 |
| Global False Discovery Rate | | < 0.1% |
| Local False Discovery Rate | | < 0.1% |
| Confidence level of protein detection | | > 95% |
| Unused ProtScore cut off | | > 1.3 |
| Number of significantly regulated proteins | Glut vs Cont | 95 |
|  | R18 vs Cont | 5 |
|  | R18 + Glut vs Cont | 17 |
|  | R18 + Glut vs Glut | 98 |
