# Supplementary material for: Proteomic analysis of cortical neuronal cultures treated with poly-arginine peptide-18 (R18) and exposed to glutamic acid excitotoxicity
Source: Mol Brain. 2019 Jul 17;12:66. doi: 10.1186/s13041-019-0486-8 (PMC6637488; doi:10.1186/s13041-019-0486-8)
Supplement: Supplementary file 2 — Table S2. Full iTRAQ proteomics data. Full iTRAQ proteomics data showing relative fold changes in protein expression and corresponding p-values. (DOCX 54 kb) [file 13041_2019_486_MOESM2_ESM.docx]

**Additional file 2: Table S2.** Full iTRAQ proteomics data showing relative fold changes in protein expression and corresponding p-values.

|  | | | | Relative to Cont | | | | | | | | | Relative to Glut | | |
| --- | --- | --- | --- | --- | --- | --- | --- | --- | --- | --- | --- | --- | --- | --- | --- |
|  | | | | **Glut (95)** | | | **R18 (5)** | | | **R18 + Glut (14)** | | | **R18 + Glut (98)** | | |
| Accession # | Gene name | Protein name | Fold-change | | *p-value* | Fold-change | | *p-value* | Fold-change | | *p-value* | Fold-change | | *p-value* |  |
| P38650 | Dync1h1 | Cytoplasmic dynein 1 heavy chain 1 | -8.873 | | 0 | -1.086 | | 0.165 | -1.109 | | 0.3695 | 8.472 | | 0 |  |
| P15146 | Map2 | Microtubule-associated protein 2 | -8.628 | | 0 | 1.138 | | 0.2091 | 1.406 | | 0.0065 | 11.482 | | 0 |  |
| P06687 | Atp1a3 | Sodium/potassium-transporting ATPase subunit alpha-3 | 1.66 | | 0.0001 | -2.128 | | 0.0319 | -1.459 | | 0.4308 | -2.355 | | 0 |  |
| P47942 | Dpysl2 | Dihydropyrimidinase-related protein 2 | 1.722 | | 0.0025 | -1.225 | | 0.4776 | 1.038 | | 0.982 | -1.69 | | 0.0029 |  |
| P82995 | Hsp90aa1 | Heat shock protein HSP 90-alpha | 1.282 | | 0.0679 | 1.038 | | 0.5187 | -1.138 | | 0.8324 | -1.446 | | 0.0442 |  |
| P63018 | Hspa8 | Heat shock cognate 71 kDa protein | -2.208 | | 0.0601 | 1.259 | | 0.592 | 1.202 | | 0.2088 | 2.559 | | 0.0034 |  |
| P12785 | Fasn | Fatty acid synthase | -3.311 | | 0.0007 | -1.117 | | 0.6532 | -1.225 | | 0.0792 | 2.704 | | 0.0459 |  |
| Q62952 | Dpysl3 | Dihydropyrimidinase-related protein 3 | 2.377 | | 0.0006 | 1.225 | | 0.3127 | 1.355 | | 0.4126 | -1.82 | | 0.0039 |  |
| Q5U300 | Uba1 | Ubiquitin-like modifier-activating enzyme 1 | -5.105 | | 0.0001 | -1.07 | | 0.6582 | -1.169 | | 0.7527 | 4.401 | | 0.0003 |  |
| P04764 | Eno1 | Alpha-enolase | 2.911 | | 0.0005 | 1.107 | | 0.4707 | 1.738 | | 0.1803 | -1.706 | | 0.0098 |  |
| Q9JHU0 | Dpysl5 | Dihydropyrimidinase-related protein 5 | 1.213 | | 0.0262 | -1.18 | | 0.904 | -1.18 | | 0.9966 | -1.459 | | 0.0405 |  |
| P05197 | Eef2 | Elongation factor 2 | -4.131 | | 0 | -1.202 | | 0.2506 | -1.343 | | 0.2981 | 3.048 | | 0.0028 |  |
| P50398 | Gdi1 | Rab GDP dissociation inhibitor alpha | 1.82 | | 0.0044 | -1.419 | | 0.2224 | -1.644 | | 0.7235 | -2.965 | | 0.002 |  |
| P05708 | Hk1 | Hexokinase-1 | 2.051 | | 0.0137 | 1.1376 | | 0.3776 | 1.086 | | 0.5298 | -1.888 | | 0.0538 |  |
| P10719 | Atp5b | ATP synthase subunit beta, mitochondrial | 2.559 | | 0.0045 | -1.117 | | 0.8748 | -1.355 | | 0.4907 | -3.342 | | 0.0009 |  |
| P61765 | Stxbp1 | Syntaxin-binding protein 1 | 1.6 | | 0.0014 | -1.107 | | 0.8187 | -1.585 | | 0.3464 | -2.466 | | 0.0001 |  |
| P15999 | Atp5a1 | ATP synthase subunit alpha, mitochondrial | 1.888 | | 0.0247 | -1.472 | | 0.2976 | -1.247 | | 0.5914 | -2.421 | | 0.0073 |  |
| P13596 | Ncam1 | Neural cell adhesion molecule 1 | 2.831 | | 0.0001 | -1.18 | | 0.1213 | -1.419 | | 0.6169 | -4.093 | | 0.0004 |  |
| P04797 | Gapdh | Glyceraldehyde-3-phosphate dehydrogenase | -11.696 | | 0 | -1.028 | | 0.4138 | -1.107 | | 0.9551 | 10.28 | | 0 |  |
| P62260 | Ywhae | 14-3-3 protein epsilon | 1.271 | | 0.0371 | -1.259 | | 0.4 | -1.009 | | 0.6834 | -1.282 | | 0.0808 |  |
| P63039 | Hspd1 | 60 kDa heat shock protein, mitochondrial | 1.49 | | 0.0204 | 1.107 | | 0.7899 | -1.047 | | 0.6988 | -1.556 | | 0.009 |  |
| P11598 | Pdia3 | Protein disulfide-isomerase A3 | 1.6144 | | 0.0209 | -1.599 | | 0.2066 | -1.247 | | 0.4028 | -1.995 | | 0.0032 |  |
| Q9JLT0 | Myh10 | Myosin-10 | -2.466 | | 0.0004 | -1.459 | | 0.3448 | -1.076 | | 0.4246 | 2.228 | | 0.0187 |  |
| P28480 | Tcp1 | T-complex protein 1 subunit alpha | -3.597 | | 0.0068 | -1.472 | | 0.2231 | -1.247 | | 0.3609 | 2.911 | | 0.0605 |  |
| P11980 | Pkm | Pyruvate kinase PKM | 1.787 | | 0.0025 | -1.009 | | 0.6284 | -1.107 | | 0.8518 | -1.941 | | 0.0016 |  |
| Q66HD0 | Hsp90b1 | Endoplasmin | 1.213 | | 0.062 | -7.3 | | 0.0984 | 1.067 | | 0.9556 | -1.138 | | 0.0476 |  |
| P21575 | Dnm1 | Dynamin-1 | -4.093 | | 0.0013 | -2.148 | | 0.1812 | -1.343 | | 0.3107 | 3.076 | | 0.0026 |  |
| Q6P502 | Cct3 | T-complex protein 1 subunit gamma | -4.787 | | 0.0004 | -1.159 | | 0.9798 | -1.169 | | 0.4019 | 4.207 | | 0.0036 |  |
| P16638 | Acly | ATP-citrate synthase | -2.399 | | 0.0176 | 1.077 | | 0.5118 | -1.38 | | 0.1501 | 1.657 | | 0.4429 |  |
| Q9ER34 | Aco2 | Aconitate hydratase, mitochondrial | -3.435 | | 0.036 | -1.028 | | 0.819 | 1.259 | | 0.2194 | 4.286 | | 0.0003 |  |
| P05065 | Aldoa | Fructose-bisphosphate aldolase A | 1.6293 | | 0.0246 | -1.659 | | 0.1425 | -1.446 | | 0.6749 | -2.355 | | 0.0114 |  |
| P48721 | Hspa9 | Stress-70 protein, mitochondrial | -6.083 | | 0.0013 | 1.159 | | 0.5277 | 1.406 | | 0.1957 | 8.318 | | 0.0003 |  |
| P06761 | Hspa5 | 78 kDa glucose-regulated protein | 2.0137 | | 0.0003 | 1.038 | | 0.8239 | 1.318 | | 0.1409 | -1.542 | | 0.0112 |  |
| Q5XIM9 | Cct2 | T-complex protein 1 subunit beta | -3.802 | | 0.0003 | 1.107 | | 0.5158 | -1.067 | | 0.203 | -1.486 | | 0.1737 |  |
| P41562 | Idh1 | Isocitrate dehydrogenase [NADP] cytoplasmic | 1.4588 | | 0.019 | 1.067 | | 0.751 | -1.019 | | 0.3093 | -1.486 | | 0.0021 |  |
| P04636 | Mdh2 | Malate dehydrogenase, mitochondrial | 2.355 | | 0.004 | -1.247 | | 0.7055 | -1.076 | | 0.6048 | -2.535 | | 0.0114 |  |
| Q63198 | Cntn1 | Contactin-1 | 1.977 | | 0.0013 | -1.117 | | 0.9479 | 1.097 | | 0.284 | -1.837 | | 0.0132 |  |
| P04642 | Ldha | L-lactate dehydrogenase A chain | 1.028 | | 0.042 | -1.786 | | 0.3591 | -1.69 | | 0.4274 | -1.675 | | 0.0083 |  |
| P04256 | Hnrnpa1 | Heterogeneous nuclear ribonucleoprotein A1 | -9.911 | | 0.0028 | -1.076 | | 0.6663 | 1 | | 0.8972 | 9.462 | | 0.0023 |  |
| P00507 | Got2 | Aspartate aminotransferase, mitochondrial | 1.2134 | | 0.1823 | -1.923 | | 0.4747 | -1.754 | | 0.1691 | -2.109 | | 0.0128 |  |
| Q9QUL6 | Nsf | Vesicle-fusing ATPase | -5.807 | | 0 | -1.117 | | 0.3188 | -1.271 | | 0.9921 | 4.656 | | 0 |  |
| Q9QXQ0 | Actn4 | Alpha-actinin-4 | 1.9055 | | 0.015 | -1.294 | | 0.5442 | 1.419 | | 0.0301 | -1.48 | | 0.4127 |  |
| P43244 | Matr3 | Matrin-3 | -3.373 | | 0.0454 | -1.5 | | 0.3077 | 1.213 | | 0.456 | 3.945 | | 0.2548 |  |
| Q9JK11 | Rtn4 | Reticulon-4 | -2.148 | | 0.0466 | 1.107 | | 0.7459 | 1.306 | | 0.2518 | 2.679 | | 0.0045 |  |
| Q9QWN8 | Sptbn2 | Spectrin beta chain, non-erythrocytic 2 | -3.105 | | 0.1053 | -2.355 | | 0.5278 | -2.466 | | 0.0477 | -1.009 | | 0.616 |  |
| P12369 | Prkar2b | cAMP-dependent protein kinase type II-beta regulatory subunit | -3.105 | | 0.0047 | -1.472 | | 0.4974 | -1.675 | | 0.5709 | 1.754 | | 0.0186 |  |
| Q68FQ0 | Cct5 | T-complex protein 1 subunit epsilon | -2.27 | | 0.0258 | 1.097 | | 0.5457 | -1.019 | | 0.5089 | 2.291 | | 0.0358 |  |
| P02770 | Alb | Serum albumin | 1.722 | | 0.0205 | -1.57 | | 0.1566 | -3.373 | | 0.0503 | -5.701 | | 0.0014 |  |
| Q9R066 | Cxadr | Coxsackievirus and adenovirus receptor homolog | -1.038 | | 0.0443 | -1.57 | | 0.4332 | -1.343 | | 0.6061 | -1.294 | | 0.0258 |  |
| P59215 | Gnao1 | Guanine nucleotide-binding protein G(o) subunit alpha | -5.152 | | 0.0183 | -1.148 | | 0.8567 | -1.33 | | 0.8705 | 3.802 | | 0.0244 |  |
| P18484 | Ap2a2 | AP-2 complex subunit alpha-2 | -2.582 | | 0.1299 | 1.159 | | 0.4561 | 1.486 | | 0.1441 | 3.698 | | 0.0092 |  |
| Q7TPB1 | Cct4 | T-complex protein 1 subunit delta | -3.945 | | 0.0013 | 1.127 | | 0.7985 | -1.067 | | 0.5181 | 3.5 | | 0.0306 |  |
| Q6P6V0 | Gpi | Glucose-6-phosphate isomerase | 1.754 | | 0.0163 | -1.318 | | 0.808 | -1.941 | | 0.3832 | -3.435 | | 0.0029 |  |
| Q9Z2L0 | Vdac1 | Voltage-dependent anion-selective channel protein 1 | 2.466 | | 0.0204 | -1.419 | | 0.4576 | -1.191 | | 0.8695 | -2.938 | | 0.0265 |  |
| P18418 | Calr | Calreticulin | 1.871 | | 0.0176 | 1.419 | | 0.2542 | 1.17 | | 0.5875 | -1.57 | | 0.0474 |  |
| P62630 | Eef1a1 | Elongation factor 1-alpha 1 | -5.441 | | 0.0012 | -1.225 | | 0.819 | -1.148 | | 0.9288 | 4.529 | | 0.0014 |  |
| P50475 | Aars | Alanine--tRNA ligase, cytoplasmic | -5.495 | | 0.0017 | -1.556 | | 0.1894 | -1.086 | | 0.6532 | 4.699 | | 0.004 |  |
| P17764 | Acat1 | Acetyl-CoA acetyltransferase, mitochondrial | 2.377 | | 0.0016 | 1.159 | | 0.3868 | 1.6 | | 0.3227 | -1.5 | | 0.0079 |  |
| O35814 | Stip1 | Stress-induced-phosphoprotein 1 | -1.047 | | 0.1947 | -1.019 | | 0.6876 | 1.009 | | 0.0642 | 1.047 | | 0.0083 |  |
| A7VJC2 | Hnrnpa2b1 | Heterogeneous nuclear ribonucleoproteins A2/B1 | -4.325 | | 0.0055 | 1.318 | | 0.2577 | 1.445 | | 0.2946 | 6.138 | | 0.0011 |  |
| Q99PF5 | Khsrp | Far upstream element-binding protein 2 | -3.908 | | 0.2961 | 1.556 | | 0.2452 | 2.07 | | 0.0468 | 8.017 | | 0.0067 |  |
| P61980 | Hnrnpk | Heterogeneous nuclear ribonucleoprotein K | -8.091 | | 0 | -1.057 | | 0.8731 | 1.077 | | 0.4615 | 8.017 | | 0.0067 |  |
| O35303 | Dnm1l | Dynamin-1-like protein | -7.519 | | 0.0462 | -1.82 | | 0.5118 | -1.419 | | 0.7224 | 8.872 | | 0 |  |
| P32551 | Uqcrc2 | Cytochrome b-c1 complex subunit 2, mitochondrial | 1.888 | | 0.0108 | -1.247 | | 0.7455 | -1.446 | | 0.2749 | 5.297 | | 0.0532 |  |
| Q66HF1 | Ndufs1 | NADH-ubiquinone oxidoreductase 75 kDa subunit, mitochondrial | 2.443 | | 0.0118 | 1.294 | | 0.6745 | -1.977 | | 0.2339 | -2.704 | | 0.0006 |  |
| P19332 | Mapt | Microtubule-associated protein tau | -7.179 | | 0.0083 | 1.077 | | 0.2359 | 1.097 | | 0.576 | -5.249 | | 0.0001 |  |
| P09951 | Syn1 | Synapsin-1 | -3.597 | | 0.009 | -1.786 | | 0.7304 | -1.096 | | 0.5875 | 3.311 | | 0.0531 |  |
| Q06647 | Atp5o | ATP synthase subunit O, mitochondrial | 2.032 | | 0.0155 | 1.406 | | 0.1826 | 1.202 | | 0.218 | -1.644 | | 0.2505 |  |
| P31044 | Pebp1 | Phosphatidylethanolamine-binding protein 1 | 2.7797 | | 0.0305 | 1.528 | | 0.5931 | 1.331 | | 0.2647 | -2.128 | | 0.1796 |  |
| Q9ESI7 | Dcx | Neuronal migration protein doublecortin | -6.139 | | 0.0048 | 1 | | 0.8287 | 1 | | 0.9864 | 6.026 | | 0.0046 |  |
| P85515 | Actr1a | Alpha-centractin | -3.565 | | 0.0408 | -1.738 | | 0.5987 | -1.528 | | 0.7481 | 2.148 | | 0.1901 |  |
| P67779 | Phb | Prohibitin | 1.6444 | | 0.0274 | -1.213 | | 0.6851 | -1.528 | | 0.5858 | -2.535 | | 0.0194 |  |
| Q9JJ54 | Hnrnpd | Heterogeneous nuclear ribonucleoprotein D0 | -5.495 | | 0.0034 | 1.202 | | 0.2000 | 1.138 | | 0.7452 | 6.252 | | 0.0112 |  |
| P62193 | Psmc1 | 26S protease regulatory subunit 4 | -22.676 | | 0.0001 | -1.419 | | 0.3232 | -1.202 | | 0.3263 | 18.197 | | 0.0005 |  |
| P69897 | Tubb5 | Tubulin beta-5 chain | -18.018 | | 0.0295 | -1.038 | | 0.8783 | -1.009 | | 0.7011 | 16.444 | | 0.0203 |  |
| Q6AYD3 | Pa2g4 | Proliferation-associated protein 2G4 | 2.1478 | | 0.1241 | 2.489 | | 0.0175 | 2.489 | | 0.0134 | 2.489 | | 0.0134 |  |
| Q5XIH7 | Phb2 | Prohibitin-2 | 3.8371 | | 0.0021 | -1.047 | | 0.7877 | 1.331 | | 0.7173 | -2.992 | | 0.0023 |  |
| Q4KM49 | Yars | Tyrosine--tRNA ligase, cytoplasmic | -4.665 | | 0 | -1.225 | | 0.1076 | -1.355 | | 0.0469 | 3.342 | | 0.0042 |  |
| P62804 | Hist1h4b | Histone H4 | -3.802 | | 0.0806 | 1.754 | | 0.1325 | 2.228 | | 0.0743 | 7.516 | | 0.0044 |  |
| P04762 | Cat | Catalase | 1.1376 | | 0.1246 | -1.556 | | 0.1149 | -1.419 | | 0.242 | -1.659 | | 0.0253 |  |
| P37285 | Klc1 | Kinesin light chain 1 | -6.024 | | 0.0268 | 1.107 | | 0.8145 | -1.191 | | 0.5122 | 5.058 | | 0.1523 |  |
| Q5RKI1 | Eif4a2 | Eukaryotic initiation factor 4A-II | -3.945 | | 0.0268 | -1.057 | | 0.8121 | -1.259 | | 0.719 | 3.02 | | 0.049 |  |
| Q3T1J1 | Eif5a | Eukaryotic translation initiation factor 5A-1 | -6.983 | | 0.032 | 1.514 | | 0.7471 | 1.5 | | 0.9944 | 9.638 | | 0.0322 |  |
| Q62829 | Pak3 | Serine/threonine-protein kinase PAK 3 | -4.365 | | 0.1069 | -2.208 | | 0.2376 | 1.406 | | 0.5015 | 5.754 | | 0.0341 |  |
| Q9EQS0 | Taldo1 | Transaldolase | -1.837 | | 0.0193 | 1.259 | | 0.7307 | 1.191 | | 0.8373 | 2.109 | | 0.0382 |  |
| O35567 | Atic | Bifunctional purine biosynthesis protein PURH | 1.9231 | | 0.1812 | 1.038 | | 0.8752 | -1.306 | | 0.6317 | -2.63 | | 0.0412 |  |
| P68255 | Ywhaq | 14-3-3 protein theta | 1.3552 | | 0.0059 | -1.086 | | 0.5518 | -1.213 | | 0.8573 | -1.64 | | 0.0044 |  |
| P19945 | Rplp0 | 60S acidic ribosomal protein P0 | -1.191 | | 0.7932 | 1.486 | | 0.0349 | -1.247 | | 0.8528 | -1.076 | | 0.7538 |  |
| P11275 | Camk2a | Calcium/calmodulin-dependent protein kinase type II subunit alpha | -6.667 | | 0.0024 | -1.419 | | 0.7004 | 1.057 | | 0.5942 | 6.918 | | 0.0017 |  |
| P27605 | Hprt1 | Hypoxanthine-guanine phosphoribosyltransferase | 1.3552 | | 0.3653 | -1.629 | | 0.6832 | 1.077 | | 0.5522 | -1.259 | | 0.0289 |  |
| P31399 | Atp5h | ATP synthase subunit d, mitochondrial | 2.754 | | 0.0329 | 1.077 | | 0.9556 | 1.556 | | 0.4116 | -1.837 | | 0.0983 |  |
| P29147 | Bdh1 | D-beta-hydroxybutyrate dehydrogenase, mitochondrial | -5.97 | | 0.0138 | -1.213 | | 0.5517 | -1.33 | | 0.6851 | 4.246 | | 0.0319 |  |
| P61265 | Stx1b | Syntaxin-1B | 1.6 | | 0.0177 | 1.225 | | 0.7322 | 1.159 | | 0.8182 | -1.419 | | 0.0232 |  |
| Q792I0 | Lin7c | Protein lin-7 homolog C OS=Rattus norvegicus | -1.614 | | 0.526 | -1.355 | | 0.2012 | -1.77 | | 0.0369 | -1.096 | | 0.3491 |  |
| F1LQ48 | Hnrnpl | Heterogeneous nuclear ribonucleoprotein L | -2.729 | | 0.0819 | -1.028 | | 0.4802 | 1.514 | | 0.8017 | 4.207 | | 0.0295 |  |
| F1LQ48 | Hnrnpl | Heterogeneous nuclear ribonucleoprotein L | -3.311 | | 0.0398 | 1.191 | | 0.6616 | 1.514 | | 0.267 | 4.92 | | 0.0127 |  |
| Q6URK4 | Hnrnpa3 | Heterogeneous nuclear ribonucleoprotein A3 | -3.945 | | 0.1787 | -1.343 | | 0.6751 | -1.169 | | 0.8649 | 3.251 | | 0.1337 |  |
| Q5M7U6 | Actr2 | Actin-related protein 2 | -1.271 | | 0.937 | -1.355 | | 0.1405 | -1.923 | | 0.0313 | -1.459 | | 0.0344 |  |
| P54290 | Cacna2d1 | Voltage-dependent calcium channel subunit alpha-2/delta-1 | 4.207 | | 0.0093 | 1.306 | | 0.775 | 1.057 | | 0.5575 | -4.018 | | 0.0052 |  |
| Q66HL2 | Cttn | Src substrate cortactin | -5.345 | | 0.0294 | 1.117 | | 0.7752 | -1.528 | | 0.2144 | 3.251 | | 0.3105 |  |
| Q8CH84 | Elavl2 | ELAV-like protein 2 | -4.405 | | 0.0911 | -1.107 | | 0.4556 | 1.225 | | 0.7267 | 5.598 | | 0.0491 |  |
| P39069 | Ak1 | Adenylate kinase isoenzyme 1 | -2.938 | | 0.0037 | 1.009 | | 0.9853 | 1.406 | | 0.7624 | 4.325 | | 0.002 |  |
| Q641Y0 | Ddost | Dolichyl-diphosphooligosaccharide--protein glycosyltransferase 48 kDa subunit | 2.466 | | 0.0211 | -1.5 | | 0.4857 | -1.148 | | 0.9084 | -2.938 | | 0.018 |  |
| P52555 | Erp29 | Endoplasmic reticulum resident protein 29 | 2.168 | | 0.0341 | -1.067 | | 0.73 | -1.432 | | 0.5763 | -3.163 | | 0.1081 |  |
| P10888 | Cox4i1 | Cytochrome c oxidase subunit 4 isoform 1, mitochondrial | 2.07 | | 0.0063 | -1.259 | | 0.9625 | -1.213 | | 0.5538 | -2.704 | | 0.0026 |  |
| P63012 | Rab3a | Ras-related protein Rab-3A | 1.271 | | 0.1795 | 1.528 | | 0.1019 | 2.109 | | 0.0478 | 1.542 | | 0.5531 |  |
| P63322 | Rala | Ras-related protein Ral-A | 2.704 | | 0.047 | -1.236 | | 0.6477 | 1.675 | | 0.4834 | -1.69 | | 0.1212 |  |
| Q62717 | Cadps | Calcium-dependent secretion activator 1 | -11.494 | | 0.0093 | -1.486 | | 0.5753 | -1.905 | | 0.6592 | 6.194 | | 0.0268 |  |
| P84092 | Ap2m1 | AP-2 complex subunit mu | -5.405 | | 0.0046 | 1.271 | | 0.5379 | 1.18 | | 0.6887 | 6.194 | | 0.0268 |  |
| P05426 | Rpl7 | 60S ribosomal protein L7 | -2.11 | | 0.0526 | 1.225 | | 0.653 | 1.077 | | 0.7063 | 2.188 | | 0.021 |  |
| Q6IFW6 | Krt10 | Keratin, type I cytoskeletal 10 | -1.722 | | 0.2367 | -2.168 | | 0.0884 | -25.84 | | 0.0168 | -16.892 | | 0.0435 |  |
| P62828 | Ran | GTP-binding nuclear protein Ran | -1.786 | | 0.0505 | 1.077 | | 0.6416 | -1.117 | | 0.6261 | 1.6 | | 0.0886 |  |
| Q62915 | Cask | Peripheral plasma membrane protein CASK | 1.3062 | | 0.0524 | 1.514 | | 0.706 | -1.419 | | 0.3692 | -1.888 | | 0.0028 |  |
| P47819 | Gfap | Glial fibrillary acidic protein | 1.9055 | | 0.0072 | 1.432 | | 0.298 | 1.69 | | 0.0411 | -1.159 | | 0.5466 |  |
| P49088 | Asns | Asparagine synthetase [glutamine-hydrolyzing] | -2.27 | | 0.0612 | -1.067 | | 0.4691 | 1.107 | | 0.4412 | 2.421 | | 0.0264 |  |
| Q63525 | Nudc | Nuclear migration protein nudC | 1 | | 0.5378 | -1.107 | | 0.7543 | 1.294 | | 0.0478 | 1.294 | | 0.0759 |  |
| P55051 | Fabp7 | Fatty acid-binding protein, brain | 4.4055 | | 0.0356 | -1.028 | | 0.8952 | 1.117 | | 0.6477 | -4.307 | | 0.0517 |  |
| P41123 | Rpl13 | 60S ribosomal protein L13 | -2.377 | | 0.051 | -1.106 | | 0.7726 | 1.17 | | 0.7438 | 2.754 | | 0.0359 |  |
| Q6IMF3 | Krt1 | Keratin, type II cytoskeletal 1 | -1.236 | | 0.367 | -4.055 | | 0.0376 | -7.943 | | 0.0424 | -6.485 | | 0.182 |  |
| Q5M7W5 | Map4 | Microtubule-associated protein 4 | -4.488 | | 0.0432 | -2.884 | | 0.3527 | -1.057 | | 0.5578 | 4.169 | | 0.0196 |  |
| P14408 | Fh | Fumarate hydratase, mitochondrial | 1.0186 | | 0.7604 | -1.225 | | 0.0458 | -1.038 | | 0.6011 | -1.067 | | 0.4492 |  |
| Q5XIF6 | Tuba4a | Tubulin alpha-4A chain | -2.679 | | 0.0303 | 1.086 | | 0.4119 | -1.047 | | 0.356 | 2.535 | | 0.1612 |  |
| P62850 | Rps24 | 40S ribosomal protein S24 | -5.754 | | 0.0722 | 1.854 | | 0.8038 | 2.334 | | 0.4094 | 14.061 | | 0.045 |  |
| Q641Y8 | Ddx1 | ATP-dependent RNA helicase DDX1 | -11.173 | | 0.0296 | -1.148 | | 0.7698 | -2.128 | | 0.4559 | 4.286 | | 0.0222 |  |
| P00388 | Por | NADPH--cytochrome P450 reductase | -1.037 | | 0.7861 | -1.107 | | 0.4592 | 1.306 | | 0.0748 | 1.343 | | 0.0452 |  |
| Q4KMA2 | Rad23b | UV excision repair protein RAD23 homolog B | 3.048 | | 0.1366 | 1.977 | | 0.2682 | 3.251 | | 0.0241 | 1.097 | | 0.6085 |  |
| O35796 | C1qbp | Complement component 1 Q subcomponent-binding protein, mitochondrial | 1.6 | | 0.0445 | 1.213 | | 0.1551 | -1.169 | | 0.5926 | -1.888 | | 0.0585 |  |
| Q71TY3 | Rps27 | 40S ribosomal protein S27 | -18.182 | | 0.0308 | -1.019 | | 0.8668 | -2.992 | | 0.1523 | 5.058 | | 0.0807 |  |
| Q62844 | Fyn | Tyrosine-protein kinase Fyn | -2.489 | | 0.058 | 1.117 | | 0.5769 | 1.148 | | 0.5379 | 2.754 | | 0.0346 |  |
| Q05175 | Basp1 | Brain acid soluble protein 1 | 1.614 | | 0.0441 | 1.343 | | 0.0316 | 1.282 | | 0.033 | -1.271 | | 0.0383 |  |
| Q5XIN6 | Letm1 | LETM1 and EF-hand domain-containing protein 1, mitochondrial | 1.459 | | 0.0172 | -3.02 | | 0.2905 | 1.259 | | 0.0291 | -1.236 | | 0.2144 |  |
| B1WBW4 | Armc10 | Armadillo repeat-containing protein 10 | -6.667 | | 0.0556 | -1.294 | | 0.5677 | -3.163 | | 0.1421 | 2.07 | | 0.0208 |  |
| F1M5N7 | Kif21b | Kinesin-like protein KIF21B | -17.857 | | 0.0051 | -1.282 | | 0.848 | 1.009 | | 0.582 | 16.444 | | 0.0048 |  |
| Q6GMN2 | Baiap2 | Brain-specific angiogenesis inhibitor 1-associated protein 2 | -90.09 | | 0.0438 | -1.33 | | 0.6067 | -1.977 | | 0.3354 | 99.083 | | 0.0175 |  |
| P62747 | Rhob | Rho-related GTP-binding protein RhoB | -1.009 | | 0.0047 | -1.038 | | 0.0094 | -1.009 | | 0.0029 | -1.009 | | 0.06 |  |
| P15087 | Cpe | Carboxypeptidase E | 1.047 | | 0.9394 | -1.019 | | 0.6562 | -1.225 | | 0.0106 | -1.294 | | 0.0442 |  |
| Q566C7 | Nudt3 | Diphosphoinositol polyphosphate phosphohydrolase 1 | 1.282 | | 0.0398 | 1.459 | | 0.0333 | 1.445 | | 0.0326 | 1.117 | | 0.1745 |  |
| P09495 | Tpm4 | Tropomyosin alpha-4 chain | -2.27 | | 0.0619 | -1.096 | | 0.4622 | -1.294 | | 0.3017 | 1.738 | | 0.0317 |  |
