# Supplementary material for: Proteomic analysis of cortical neuronal cultures treated with poly-arginine peptide-18 (R18) and exposed to glutamic acid excitotoxicity
Source: Mol Brain. 2019 Jul 17;12:66. doi: 10.1186/s13041-019-0486-8 (PMC6637488; doi:10.1186/s13041-019-0486-8)

**Additional file 3: Table S3.** PANTHER gene-ontology functional categorization of DEPs significantly regulated by R18 treatment alone (R18 vs Cont), glutamic acid exposure (Glut vs Cont), and R18 pre-treatment with glutamic acid exposure (R18 + Glut vs Glut). *Note:* proteins may have multiple functions, and as such, the total number of proteins in each category may be greater than the sum of DEPs across each treatment group.

| **Cellular Component** | | | | | | | |
| --- | --- | --- | --- | --- | --- | --- | --- |
|  |  | **R18 vs Cont** | | | **R18 + Glut vs Glut** | | |
|  | **Category name (Accession)** | **# genes** | **% of gene hit against total # genes** | **% of gene hit against total # Function hits** | **# genes** | **% of gene hit against total # genes** | **% of gene hit against total # Function hits** |
| cell (GO:0005623) | | 3 | 60.00% | 75.00% | 47 | 46.50% | 54.70% |
| organelle (GO:0043226) | | 1 | 20.00% | 25.00% | 21 | 20.80% | 24.40% |
| protein-containing complex (GO:0032991) | | 0 | - | - | 13 | 12.90% | 15.10% |
| membrane (GO:0016020) | | 0 | - | - | 3 | 3.00% | 3.50% |
| extracellular region (GO:0005576) | | 0 | - | - | 1 | 1.00% | 1.20% |
| cell junction (GO:0030054) | | 0 | - | - | 1 | 1.00% | 1.20% |
| **Molecular Function** | | | | | | | |
| catalytic activity (GO:0003824) | | 2 | 40.00% | 40.00% | 47 | 46.50% | 45.20% |
| binding (GO:0005488) | | 1 | 20.00% | 20.00% | 43 | 42.60% | 41.30% |
| transporter activity (GO:0005215) | | 1 | 20.00% | 20.00% | 7 | 6.90% | 6.70% |
| structural molecule activity (GO:0005198) | | 1 | 20.00% | 20.00% | 4 | 4.00% | 3.80% |
| translation regulator activity (GO:0045182) | | 0 | - | - | 1 | 1.00% | 1.00% |
| molecular transducer activity (GO:0060089) | | 0 | - | - | 1 | 1.00% | 1.00% |
| transcription regulator activity (GO:0140110) | | 0 | - | - | 0 | - | - |
| molecular function regulator (GO:0098772) | | 0 | - | - | 1 | 1.00% | 1.00% |
| **Biological Process** | | | | | | | |
| cellular process (GO:0009987) | | 1 | 20.00% | 20.00% | 40 | 39.60% | 33.10% |
| metabolic process (GO:0008152) | | 2 | 40.00% | 40.00% | 33 | 32.70% | 27.30% |
| localization (GO:0051179) | | 1 | 20.00% | 20.00% | 18 | 17.80% | 14.90% |
| multicellular organismal process (GO:0032501) | | 0 | - | - | 11 | 10.90% | 9.10% |
| biological regulation (GO:0065007) | | 1 | 20.00% | 20.00% | 9 | 8.90% | 7.40% |
| response to stimulus (GO:0050896) | | 0 | - | - | 7 | 6.90% | 5.80% |
| developmental process (GO:0032502) | | 0 | - | - | 0 | - | - |
| biological adhesion (GO:0022610) | | 0 | - | - | 0 | - | - |
| reproduction (GO:0000003) | | 0 | - | - | 0 | - | - |
| cellular component organization or biogenesis (GO:0071840) | | 0 | - | - | 3 | 3.00% | 2.50% |


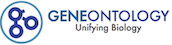

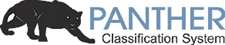

Supplement: Supplementary file 3 — Table S3. PANTHER gene-ontology functional categorization. PANTHER gene-ontology functional categorization of DEPs significantly regulated by R18 treatment alone (R18 vs Cont), glutamic acid exposure (Glut vs Cont), and R18 pre-treatment with glutamic acid exposure (R18 + Glut vs Glut). Note: proteins may have multiple functions, and as such, the total number of proteins in each category may be greater than the sum of DEPs across each treatment group. (DOCX 49 kb) [file 13041_2019_486_MOESM3_ESM.docx]
