# Supplementary material for: Proteomic analysis of cortical neuronal cultures treated with poly-arginine peptide-18 (R18) and exposed to glutamic acid excitotoxicity
Source: Mol Brain. 2019 Jul 17;12:66. doi: 10.1186/s13041-019-0486-8 (PMC6637488; doi:10.1186/s13041-019-0486-8)

**Additional file 4: Table S4.** Cytoscape quantitative analysis of STRING data cluster strength for 73 shared DEPs across Glut and R18 + Glut treatment groups.

| **Cluster** | | **#Nodes** | | **Density** | | **Internal weight** | | **External weight** | | ***p-*value** | | **Members** | |
| --- | --- | --- | --- | --- | --- | --- | --- | --- | --- | --- | --- | --- | --- |
| "Mitochondrial Respiration" | 55 | | 0.802 | | 1191 | | 26 | | 0 | | Ndufv2 Atp5h Atp5i Atp5o Uqcrh Ndufb3 Ndufc2 Ndufa7 Ndufa6 Ndufb9 Ndufs4 Ndufb5 Ndufs3 Ndufs1 Ndufa5 Ndufa12 Atp5b Ndufb7 Uqcrc2 Uqcrfs1 Atp5d Cox4i1 Cyc1 Ndufa9 Ndufa10 Ndufb10 Ndufb8 Ndufs8 Ndufa2 Ndufv1 Mdh2 Atp5a1 ND1 Uqcrc1 Ndufb2 Ndufb6 Ndufs7 ND6 ND4 ND2 CYTB ND3 ND5 Vdac1 Ndufa11 Atp5c1 Atp5g3 Atp5l Ndufs2 Ndufc1 Ndufa8 Aco2 Atp5f Uqcrq Atp5e | |  |
| "Proteasome & Protein Synthesis" | 43 | | 0.7586 | | 685 | | 33 | | 0 | | Psmd3 Psma7 Psmb3 rCG_63409 Psmb8 Psma1 Usp14 Psmb4 Psmb6 Psmb5 Adrm1 Psmb7 Psmb1 Psmd7 Psmd8 Psmc1 Psmc6 Psmd13 Psmc4 Psmc3 Psma4 Psmd12 Psmd14 Psma5 Psmd4 Psma3 Psmd6 Psmd1 Psmc5 Psmc2 Psmd2 Psma6 Psmb2 Cct8 Psmd11 Cct4 Hnrpd Psma2 Cct5 Cct7 Tcp1 Cct3 Cct2 | |  |
| "Axonal growth & neuronal differentiation" | 11 | | 0.8545 | | 47 | | 16 | | 4.54x10⁻⁵ | | Ap2b1 Syt1 Vamp2 Dnm1 Ap2a1 Ap2a2 Itsn1 Arrb1 Ap2m1 Ap2s1 Dpysl2 | |  |
| "Transmembrane trafficking" | 10 | | 0.6889 | | 31 | | 21 | | 5.039x10⁻³ | | Syt1 Vamp2 Dnm1 Nsf Stx1b Stxbp1 Stx1a Snap25 Cplx1 Cadps | |  |
| "ER proteostasis" | 8 | | 0.5 | | 14 | | 14 | | 0.0205 | | Phb2 Hsp90b1 Hspa9 Pdia3 Calr Hspa5 Rpn2 Phb | |  |
| "Glycolysis & Carbohydrate Metabolism" | 7 | | 0.7143 | | 15 | | 7 | | 0.0024 | | Gpi Eno1 Pkm Taldo1 Aldoa Gapdh Alb | |  |
| "RNA trafficking and processing" | 4 | | 0.8333 | | 5 | | 4 | | 0.0257 | | Hnrnpa2b1 Hnrnpa1 Hnrnpa3 Hnrnpk | |  |
| "Mitochondrial fatty acid synthesis" | 4 | | 0.5 | | 3 | | 2 | | 0.0432 | | Fasn Acaca Acat1 Bdh1 | |  |


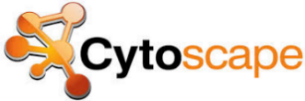

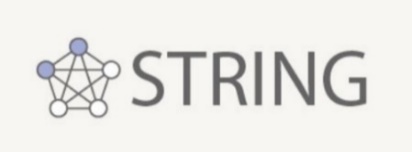

Supplement: Supplementary file 4 — Table S4. Cytoscape quantitative analysis. Cytoscape quantitative analysis of STRING data cluster strength for 73 shared DEPs across Glut and R18 + Glut treatment groups. (DOCX 56 kb) [file 13041_2019_486_MOESM4_ESM.docx]
