# Supplementary material for: Proteomic analysis of cortical neuronal cultures treated with poly-arginine peptide-18 (R18) and exposed to glutamic acid excitotoxicity
Source: Mol Brain. 2019 Jul 17;12:66. doi: 10.1186/s13041-019-0486-8 (PMC6637488; doi:10.1186/s13041-019-0486-8)
Supplement: Supplementary file 5 — Table S5. Quantitative data and full gene list of KEGG pathway analysis. Quantitative data and full gene list of KEGG pathway analysis of 73 shared DEPs across Glut and R18 + Glut treatment groups, with details provided on term ID, overserved gene count vs. background gene count, and FDR. (DOCX 17 kb) [file 13041_2019_486_MOESM5_ESM.docx]

**Additional file 5: Table S5.** Quantitative data and full gene list of KEGG pathway analysis of 73 shared DEPs across Glut and R18 + Glut treatment groups.

| **#term ID** | **Term description** | **Observed gene count** | **Background gene count** | **% pathway** | **False discovery rate** |
| --- | --- | --- | --- | --- | --- |
| rno03050 | Proteasome | 34 | 46 | 73.9 | 1.67E-50 |
| rno00190 | Oxidative phosphorylation | 52 | 130 | 40.0 | 7.64E-67 |
| rno05012 | Parkinson's disease | 52 | 134 | 38.8 | 1.41E-66 |
| rno05010 | Alzheimer's disease | 47 | 164 | 28.7 | 5.49E-55 |
| rno05016 | Huntington's disease | 50 | 181 | 27.6 | 8.56E-58 |
| rno04932 | Non-alcoholic fatty liver disease (NAFLD) | 35 | 146 | 23.9 | 3.27E-38 |
| rno04723 | Retrograde endocannabinoid signalling | 34 | 144 | 23.6 | 5.36E-37 |
| rno04714 | Thermogenesis | 52 | 221 | 23.5 | 3.08E-57 |
| rno04721 | Synaptic vesicle cycle | 14 | 60 | 23.3 | 4.77E-15 |
| rno00072 | Synthesis and degradation of ketone bodies | 2 | 9 | 22.2 | 0.0157 |
| rno00061 | Fatty acid biosynthesis | 2 | 14 | 14.3 | 0.031 |
| rno04961 | Endocrine and other factor-regulated calcium reabsorption | 7 | 52 | 13.5 | 2.95E-06 |
| rno04260 | Cardiac muscle contraction | 10 | 75 | 13.3 | 1.01E-08 |
| rno01210 | 2-Oxocarboxylic acid metabolism | 2 | 17 | 11.8 | 0.001 |
| rno00620 | Pyruvate metabolism | 4 | 35 | 11.4 | 0.0017 |
| rno00630 | Glyoxylate and dicarboxylate metabolism | 3 | 28 | 10.7 | 0.0106 |
| rno00020 | Citrate cycle (TCA cycle) | 3 | 29 | 10.3 | 0.0111 |
| rno00030 | Pentose phosphate pathway | 3 | 29 | 10.3 | 0.0111 |
| rno04130 | SNARE interactions in vesicular transport | 3 | 31 | 9.7 | 0.0121 |
| rno01230 | Biosynthesis of amino acids | 7 | 73 | 9.6 | 2.25E-05 |
| rno04962 | Vasopressin-regulated water reabsorption | 4 | 42 | 9.5 | 0.0029 |
| rno05169 | Epstein-Barr virus infection | 19 | 206 | 9.2 | 1.04E-13 |
| rno01200 | Carbon metabolism | 10 | 112 | 8.9 | 3.22E-07 |
| rno00010 | Glycolysis / Gluconeogenesis | 5 | 59 | 8.5 | 0.001 |
| rno00510 | N-Glycan biosynthesis | 3 | 48 | 6.3 | 0.0318 |
| rno04911 | Insulin secretion | 5 | 82 | 6.1 | 0.0035 |
| rno04918 | Thyroid hormone synthesis | 4 | 68 | 5.9 | 0.0121 |
| rno01212 | Fatty acid metabolism | 3 | 51 | 5.9 | 0.0354 |
| rno01100 | Metabolic pathways | 68 | 1240 | 5.5 | 3.56E-38 |
| rno04141 | Protein processing in endoplasmic reticulum | 7 | 157 | 4.5 | 0.0019 |
| rno04066 | HIF-1 signalling pathway | 4 | 99 | 4.0 | 0.0354 |
| rno04144 | Endocytosis | 7 | 252 | 2.8 | 0.0182 |
